# Visfatin (NAMPT) expression in human placenta cells in normal and pathological conditions and its hormonal regulation in trophoblast JEG-3 cells

**DOI:** 10.1371/journal.pone.0310389

**Published:** 2024-09-18

**Authors:** Monika Dawid, Patrycja Kurowska, Piotr Pawlicki, Małgorzata Kotula–Balak, Tomasz Milewicz, Joelle Dupont, Agnieszka Rak

**Affiliations:** 1 Laboratory of Physiology and Toxicology of Reproduction, Institute of Zoology and Biomedical Research, Jagiellonian University in Krakow, Kraków, Poland; 2 Doctoral School of Exact and Natural Sciences, Jagiellonian University in Kraków, Kraków, Poland; 3 Department of Animal Anatomy and Preclinical Sciences, University Centre of Veterinary Medicine JU-UA, University of Agriculture in Krakow, Kraków, Poland; 4 Center of Experimental and Innovative Medicine, University of Agriculture in Krakow, Kraków, Poland; 5 Department of Gynecological Endocrinology, Jagiellonian University Medical College, Kraków, Poland; 6 INRAE, UMR0085, Unité Physiologie de la Reproduction et des Comportements, Nouzilly, France; Virgen Macarena University Hospital, School of Medicine, University of Seville, SPAIN

## Abstract

Visfatin is an adipokine involved in energy metabolism, insulin resistance, inflammation, and female reproduction. Due to limited data about its action in the human placenta, the aims of our studies included the analysis of visfatin expression and immunolocalization in trophoblast cell lines JEG-3 and BeWo as well as in human placentas from normal and pathological pregnancies. Moreover, we also checked the hormonal regulation of visfatin levels and the molecular mechanism of observed changes in JEG-3 cells. Cell culture and placental fragments collection along with statistical analysis were performed using standard laboratory procedures also described in our previous papers. We demonstrated an increased gene and protein expression of visfatin in JEG-3, BeWo cells, while variable expression in maternal and fetal parts of normal/ pathological pregnancy placentas. In addition, the immunolocalization of visfatin was observed in the cytoplasm of both cell lines, the capillary epithelium of the maternal part and syncytiotrophoblasts of the placental fetal part; in all tested pathologies, the signal was also detected in decidual cells. Furthermore, we demonstrated that hormones: progesterone, estradiol, human chorionic gonadotropin, and insulin increased the visfatin levels in JEG-3 cells with the involvement of specific signaling pathways. Taken together, differences in the expression and localization of visfatin between normal and pathological placentas suggested that visfatin may be a potential marker for the diagnosis of pregnancy disorders. In addition, we found that placental levels of visfatin can be regulated by hormones known to modulate the function of placental cells.

## Introduction

Visfatin was first isolated in 1994 from human peripheral blood lymphocytes as pre-B cell colony enhancing factor (PBEF) [1]. Later studies showed its high homology with the enzyme nicotinamide phosphoribosyltransferase (NAMPT) [2], while Fukuhara et al. [3] named the newly discovered adipokine visfatin because its highest expression was described in visceral adipose tissue. It is worth mentioning that the work in which the name visfatin was first used was finally withdrawn by the authors themselves due to controversy regarding the insulinomimetic effect of the hormone they studied. Nevertheless, the name visfatin, due to its highest expression of protein in visceral fat, functions and is one of the most widely used to this day [3]. Thus, visfatin/PBEF/NAMPT is a 52 kDa protein, whose human gene is located on the long arm of chromosome 7 between 7q22.1 and 7q31.33 [4]. The intracellular form of visfatin (iNAMPT) regulates the level of oxidized nicotinamide adenine dinucleotide (NAD+), a key coenzyme in the redox reaction in all living cells, while the extracellular form (eNAMPT) behaves like a cytokine in response to cellular stress or inflammatory processes [5]. Visfatin expression has been investigated in the cytoplasm and nucleus of many cells, with the highest levels observed in bone marrow, the liver, and muscles, but visfatin is also present in visceral fat, the brain, the kidneys, the spleen, and the testes, as well as the placenta, fetal membranes, and the myometrium [6, 7].

The expression of visfatin in many tissues indicates its pleiotropic effect in organisms; it may regulate energy metabolism and inflammatory, cardiovascular, or reproductive processes, as well as the course of pregnancy [8]. Interestingly, a visfatin-specific receptor has not been identified, but some studies suggest that visfatin exerts its effect through toll-like receptor 4 (TLR4) or enzymatic activity of NAMPT [9]. Nevertheless, most studies indicate that visfatin can control cellular processes by binding to the insulin receptor (INSR) [10]. In above mentioned rejected report of an insulinomimetic effect, was studied that visfatin acts like insulin and may increase glucose uptake in mouse 3T3-L1 adipocytes and rat L6 myocytes and suppress glucose release in rat hepatoma cells [3]. Moreover, visfatin may regulate insulin secretion, phosphorylation of INSR, or intracellular signaling, as well as the expression of genes related to mouse β-cell function [10]. It is also well known that visfatin plasma levels increase in hyperglycemic states, insulin resistance, and obesity, as well as in physiological pregnancy, while obesity and obesity-related pathologies cause a further increase [11]. Furthermore, Nampt ^+/−^ heterozygous female mice show impaired glucose tolerance and significantly reduced insulin secretion in intraperitoneal glucose tolerance tests [12]. It has been shown that in human monocytes, visfatin stimulated the production of pro-inflammatory cytokines, such as tumor necrosis factor-α (TNF-α) or interleukin-6 (IL-6) [13]. Likewise, visfatin increased the proliferation, migration, and formation of capillary-like tubes in human umbilical vein endothelial cells during pregnancy, suggesting a potential role of visfatin during fetal development [14]. It is well known that obesity leads to numerous pregnancy pathologies, such as intrauterine growth restriction (IUGR), preeclampsia (PE), or gestational diabetes mellitus (GDM), and the plasma levels of visfatin are higher in these pregnancy pathologies than in healthy pregnant women [15–17] however, data on the expression and immunolocalization of visfatin in human placenta cells are limited.

Therefore, the goal of the present study was to investigate visfatin gene and protein expression and visfatin localization in human trophoblast JEG-3 and BeWo cells, as well as in placental explants from normal pregnancies and those complicated by IUGR, PE, or GDM. In the next part of our study, we investigated the regulation of placental visfatin levels by pregnancy hormones such as progesterone (P_4_), estradiol (E_2_), human chorionic gonadotropin (hCG), and insulin (INS) in JEG-3 cells. The above-mentioned hormones are important at each stage of pregnancy, P_4_ stimulates the decidualization of human embryonic stem cells *in vitro* and promotes embryo implantation [18]. In turn, E_2_ promotes embryo implantation, stimulates growth and differentiation of the endometrium, and induces vasodilation of the uterine and placental arteries [18]. On the other hand, hCG, which has autocrine and paracrine effects, is a pleiotropic factor; it is a primary marker of pregnancy diagnosis and stimulates the production of P_4_ and the *in vitro* differentiation of human cytotrophoblasts into syncytiotrophoblasts [19]. The INS inhibits glucose production and stimulates glucagon production, and research shows that insulin resistance, and thus the need for insulin, increases significantly after the 20^th^ week of pregnancy [20]. Moreover, unregulated levels of P_4_, E_2_, hCG, or INS can lead to the development of IUGR, PE, or GDM pathologies [21, 22].

## Methods

### *In vitro* placental cell culture and experimental protocol

The JEG-3 (cat. no. HTB-36; American Type Culture Collection, USA) and BeWo (cat. no. CCL-98; American Type Culture Collection, USA) placental cell lines are a good model for testing placental function in *in vitro* conditions which we also showed in our previous research [23, 24]. The JEG-3 cells are derived from the extracellular cytotrophoblast (EVT), while the BeWo cells are obtained from the villous cytotrophoblast (VCT), which undergoes syncytialization upon forskolin treatment [25]. Microarray analysis showed that approximately 2700 genes are expressed differently between these cell lines, suggesting that these cells are suitable for specific experiments [26]. JEG-3 cells were cultured in DMEM/ F12 medium without phenol red, supplemented with 10% fetal bovine serum (FBS), while BeWo cells were cultured in DMEM/F12 medium without phenol red but supplemented with 10% FBS and 1% L-glutamine in 5% CO_2_/95% air at 37°C [24].

#### Experiment 1

After reaching approximately 80% confluency, cells from both lines were seeded and cultured in the previous media at a density of 4 x 10^3^ cells/well on 96-well plates to measure the levels of visfatin gene and protein expression as well as the visfatin concentration in the culture medium (n = 3). After 24 h of cell seeding, the medium was changed, and cells were incubated for 24, 48, and 72 h in DMEM/F12 with 1% FBS for JEG-3 cells or in DMEM/F12 with 10% FBS for BeWo cells [44]. After 24, 48, 72 h of cell incubation, JEG-3 and BeWo cells were stored at -70°C or -20°C for further real-time polymerase chain reaction (qRT-PCR) and Western blot analysis, respectively. In addition, cells were seeded at a density of 2 x 10^4^ cells/well for 48 h of incubation on coverslips in 24-well plates to determine visfatin immunolocalization (n = 3). Additionally, human placental slides purchased commercially (cat. no. T2234200, BioChain, USA) were used to study visfatin immunolocalization (n = 3).

**Fig 1:**
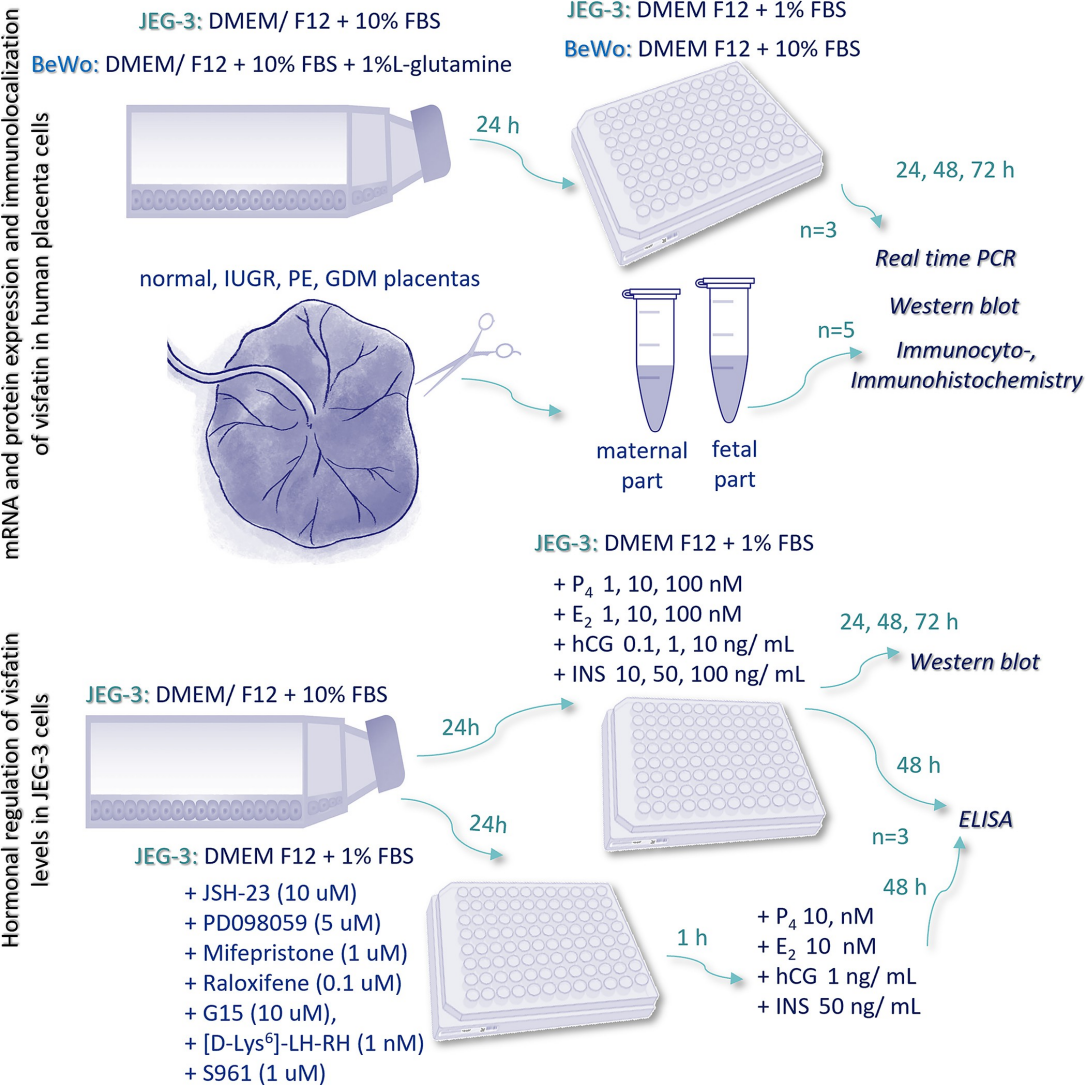
The scheme of conducted experiments. IUGR—intrauterine growth restriction, PE—preeclampsia, GDM—gestational diabetes mellitus; P4—progesterone; E2—estradiol; hCG—human chorion gonadotropin; INS—insulin; mifepristone—membrane (mPR)/ nuclear (PR) P_4_ receptor antagonist; G15—membrane E_2_ receptor (GPR30) antagonist; raloxifene—nuclear E_2_ receptor (ER) antagonist; [D-Lys^6^]-LH-RH—hCG receptor (LHCGH) antagonist; S961—INS receptor (INSR) antagonist; PD098059—inhibitor of extracellular signal-regulated kinase (ERK1/2); JSH-23—inhibitor of transcription factor NF kappa B (NF-κB); FBS—fetal bovine serum.

Fragments of human term placentas (38–40 weeks of pregnancy) were collected from 26^th^ July 2023 to 1^st^ October 2023 from healthy women (n = 5) and those diagnosed by a gynecologist with PE (n = 5), IUGR (n = 5), or GDM (n = 5) from the Clinical Department of Gynecological Endocrinology, University Hospital, Kraków, in connection with the decision of the Bioethics Committee no: 1072.6120.252.2022. All study participants (women 20–40 years old with correct weight, and BMI<25 kg/m^2^), gave informed, written consent to participate in the study. Within 30 min after delivery, the material was transported in PBS with 100 IU/mL penicillin and 100 g/mL streptomycin to the laboratory, where placentas were rinsed three times with fresh PBS containing penicillin and streptomycin. Afterward, the fragments were divided into maternal and fetal parts (50 mg) and frozen at -70°C and -20°C for further qRT-PCR and Western blot analysis, respectively (n = 5) (Fig 1). To examine the cellular immunolocalization of visfatin, the placenta fragments were fixed in formaldehyde for further analysis (n = 3).

#### Experiment 2

JEG-3 cells were treated with E_2_ at doses of 1, 10, and 100 nM (cat. no. E2257, Sigma-Aldrich, USA), with P_4_ at doses of 1, 10, and 100 nM (cat. no. P0130, Sigma-Aldrich, USA), with hCG at doses of 0.1, 1, and 10 ng/mL (cat. no. C0434, Sigma-Aldrich, USA), or with INS at doses of 10, 50, and 100 ng/mL (cat. no. 15523, Sigma-Aldrich, USA) for 24, 48, or 72 h (see Experimental protocol, Fig 1) (n = 3). Following incubation, culture media were harvested and centrifuged at 1000 × g for 10 min at 4°C, and the supernatants were collected and stored at -20°C for analysis of visfatin concentration, while the cells were lysed using Laemmli buffer and then stored at -20°C for analysis of visfatin protein expression.

#### Experiment 3

JEG-3 cells were first treated for 1 h with antagonists for transcription factor NF kappa B (NF-κB): JSH-23 at a dose of 10 uM (cat. no. J4455, Sigma-Aldrich, USA), extracellular signal-regulated kinase (ERK1/2): PD098059 at a dose of 5 uM (cat. no. 1213, Tocris Bioscience), membrane (mPR)/nuclear (PR) P_4_ receptors: mifepristone at a dose of 1 uM (cat. no. 475838, Millipore, USA), nuclear estrogen receptor (ER): raloxifene at a dose of 0.1 uM (cat. no. PHR1852, Sigma-Aldrich, USA), G protein-coupled receptor 30 (GPR30): G15 at a dose of 10 uM (T7389-2, TargetMol, USA), human chorionic gonadotrophin receptor (LHCGH): [D-Lys^6^]-LH-RH at a dose of 1 nM (cat. no. SCP0180, Sigma-Aldrich, USA), and insulin receptor (INSR): S961 at a dose of 1 uM (cat. no. 051–86, Phoenix Pharmaceuticals, USA) (n = 3). After that, the hormones E_2_ (10 nM), P_4_ (10 nM), hCG (1 ng/mL), and INS (50 ng/mL) were added for 48 h. The doses of all tested hormones and antagonists were selected based on the literature and our preliminary results [27–29] (see Experimental protocol, Fig 1). Following incubation, the culture media were harvested and centrifuged at 1000 × g for 10 min at 4°C, and the supernatants were collected and stored at -20°C for analysis of visfatin concentration.

### qRT-PCR

Total RNA isolation and reverse transcription (1 h, 37°C) were carried out on JEG-3 or BeWo cells using the TaqMan Gene Expression Cells-to-CT kit (cat. no. A35374, Thermo Fisher Scientific, USA), while placental tissues were analyzed with TRIzol (cat. no. 15596026, Thermo Fisher Scientific, USA) and a reverse transcriptase assay (cat. no. 04896866001, Roche, Switzerland) following the manufacturer’s protocol. The resulting cDNA was analyzed using the StepOnePlus Real-Time PCR System (Applied Biosystems; Thermo Fisher Scientific, USA) and the TaqMan Gene Expression Assay for visfatin (cat. no. Hs00237184, RefSeq NM_005746.2, Applied Biosystems; Thermo Fisher Scientific, USA) in combination with TaqMan Gene Expression Master Mix containing the reference dye ROX (cat. no. 399002, Applied Biosystems; Thermo Fisher Scientific, USA). The reaction was performed under the following cycle conditions: 50°C for 2 min, 95°C for 10 min, 40 cycles of 95°C for 15 s, and 60°C for 60 s. Gene expression was normalized using the geometric mean of three reference genes: GAPDH (cat. no. Hs02786624; RefSeq NM_001256799.2, Thermo Fisher Scientific, USA), TBP (cat. no. Hs00920495_m1; RefSeq NM_001172085.1, Thermo Fisher Scientific, USA), and YWHAZ (cat. no. Hs01122445_g1; RefSeq NM_09111, Thermo Fisher Scientific, USA) using the 2^-ΔCt^ method [30].

### Western blot method

Western blotting and quantification were performed as previously described [23]. Briefly, equivalent amounts of lysate were applied (~20–40 μg protein/lane), and then the proteins were separated by hand-casting 10% polyacrylamide gels and transferred into a PVDF membrane using Trans-Blot Turbo Mini PVDF Transfer Packs (Bio-Rad Laboratories, Inc.). In the next step, the membranes were blocked for 1 h in 0.02 M Tris-buffered saline with 5% bovine serum albumin (BSA) and 0.1% Tween-20 at room temperature (RT) and then incubated overnight at 4°C with primary antibody for anti-visfatin (1:500, cat. no. ab233294, Abcam, UK) which validation using blocking peptide was described in our previous paper [31]. Subsequently, the membranes were rinsed in Tris-buffered saline containing 0.1% Tween-20 and incubated for 1 h at RT with a horseradish peroxidase-conjugated secondary anti-rabbit antibody (1:1000, cat. no. 7074, Cell Signaling, USA). The loading control contained β-actin (ACTB) (1:1000, cat. no. A5316, Sigma-Aldrich, USA) and secondary anti-mouse antibody (1:1000, cat. no. 7074, Cell Signaling, USA), while the chemiluminescence signal was detected using an HRP substrate (cat. no. WBKLS0500, Millipore, USA) and visualized by the Chemidoc™ XRS+ System (BioRad, USA). Each band was quantified by densitometry and ImageJ software (version 1.51, National Institutes of Health, USA).

### Immunocytochemistry and immunohistochemistry analysis

JEG-3 and BeWo cells were fixed with 2% formaldehyde freshly prepared from paraformaldehyde and permeabilized with 0.1% Triton X-100 in Tris-buffered saline (TBS; 0.05 M Tris-HCl plus 0.15 M NaCl, pH 7.6). Then, the cells were immersed in 3% H_2_O_2_ for 10 min at RT, blocked in 5% goat serum for 30 min (Sigma-Aldrich, USA), and incubated overnight at 4°C with primary antibodies against visfatin (1:100, cat. no. ab233294, Abcam, UK). Subsequently, goat anti-rabbit biotinylated antibody (1:400, cat. no. BA-1000-1.5, Vector, USA) and avidin biotinylated horseradish peroxidase complex (1:500, cat. no. PK- 6100, VECTASTAIN Elite ABC Kits, USA) were applied at RT, and the bound antibody was visualized with a chromogenic substrate containing 0.05% 3,3′-diaminobenzidine (DAB) (cat. no. D4293, Sigma-Aldrich, USA). In control slips, the primary antibody was omitted or replaced by irrelevant IgG. Afterward, the material was washed and counterstained with Mayer’s hematoxylin at RT for 10 s and finally mounted using DPX mounting media (cat. no. 44581, Sigma-Aldrich, USA). Serial sections stained for visfatin were examined by a blinded observer with a Leica DMR microscope using a 20× objective (Leica Microsystems, Wetzlar, Germany). Also, prepared slides with placental tissues obtained from the hospital or purchased commercially were immersed in 10 mM citrate buffer (pH 6.0) and heated in a microwave oven (2×5 min, 700 W). Thereafter, the procedure was the same as described above for immunocytochemistry.

### ELISA kit

Visfatin levels in the culture medium were determined using the commercially available human ELISA kit (cat. no. EH0651, Fine Test, China) following the manufacturer’s protocol. The sensitivity of the visfatin assay was 0.188 ng/mL, and the intra- and inter-assay precision was less than 5.12% and 5.82%, respectively. Absorbance values were measured at 450 nm using a Varioskan LUX Multimode Microplate Reader and SkanIt software 6.1.1 (Thermo Fisher Scientific, Waltham, MA, USA).

### Statistical analysis

All experiments were repeated at least three (n = 3), while and the data were analyzed using one or two-way ANOVA followed by Tukey’s HSD multiple range test in GraphPad PRISM (version 8.0.1, GraphPad Software, USA). The distribution of normality was checked with a Shapiro–Wilk test. Obtained data were presented as the mean ± SEM. Statistical significance is indicated by different letters (p < 0.05), with a < b < c < d < e < f < g; identical letters indicate no significant difference.

## Results

### Gene and protein expression of visfatin in JEG-3 and BeWo cells as well as its immunolocalization

**Fig 2:**
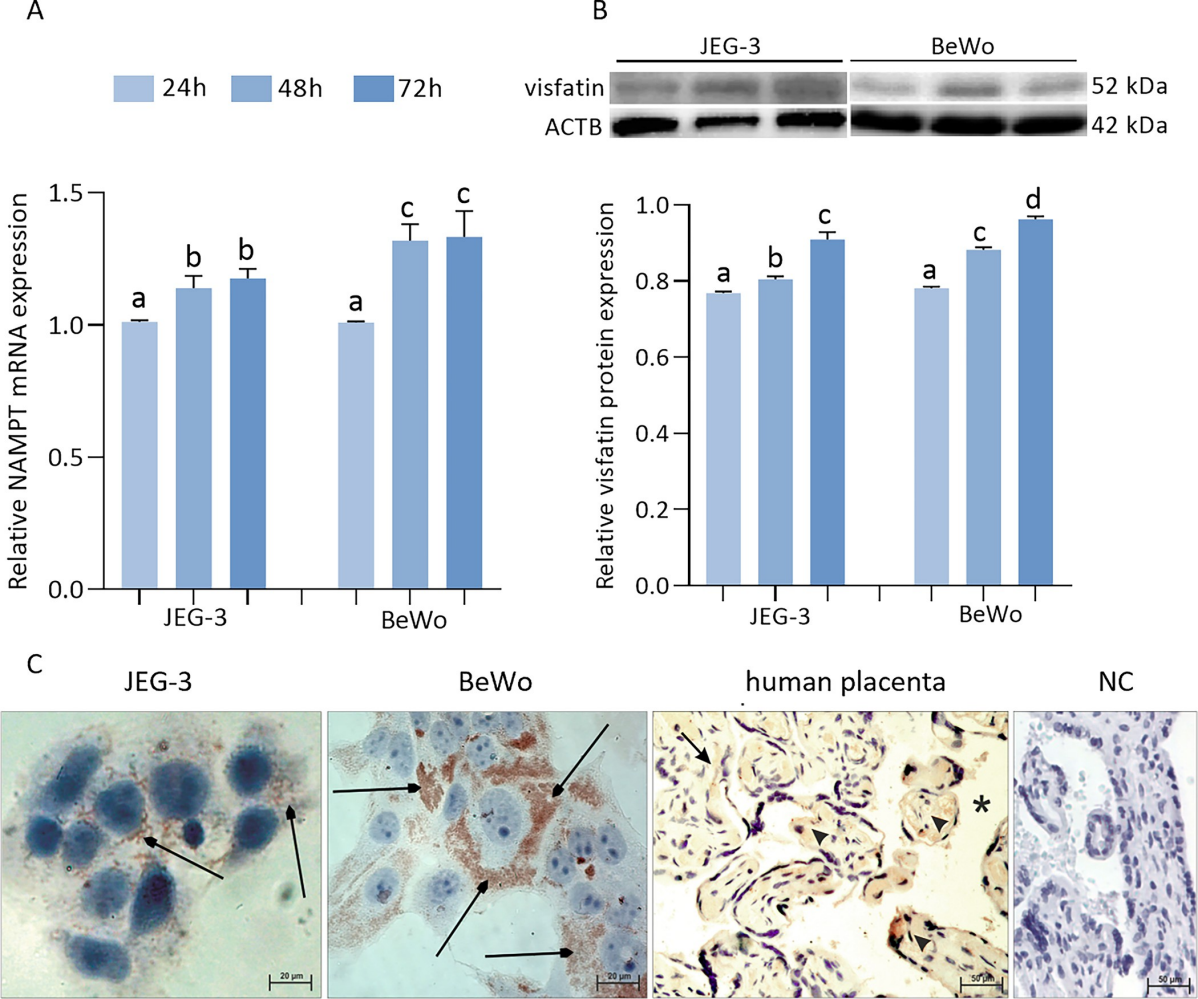
Gene (A) and protein expression (B) of visfatin (p < 0.05) as well as its immunolocalization in JEG-3, BeWo cell lines and commercially human term placenta slides (C) (scale bar is 20 μm). Immunolocalization: cytoplasm of cells (arrows), villous cells (arrowheads), blood spaces surrounding the villi (asterisks). Fig 2B shows data from multiple blot images. The relative gene expression of visfatin was examined by qRT-PCR then the obtained results were normalized using the geometric mean of reference gene expression (GAPDH, TBP, YWHAZ) due to the comparative cycle threshold method. The protein expression of visfatin was detected by Western blot, and protein lanes were densitometrically measured and shown as the ratio relative to ACTB expression. Statistical analysis was shown using ANOVA followed by Tukey’s HSD multiple range test (mean ± SEM, p < 0.05; n = 3). ACTB—β-actin; NC—negative control.

The obtained data indicated a time-dependent increase in the expression of NAMPT/visfatin mRNA and protein in both JEG-3 cells (1, 1.14 ± 0.13, 1.19 ± 0.07) and BeWo cells (1, 1.24 ± 0.02, 1.30 ± 0.12) after 24, 48, and 72 h, respectively (Fig 2A and 2B, p < 0.05). We observed that visfatin gene and protein expression were higher in BeWo cells than in JEG-3 cells after 48 and 72 h of cell incubation (p < 0.05).

The immunostaining of DAB with hematoxylin contrast staining revealed the presence of visfatin in both trophoblast JEG-3 and BeWo cells (Fig 2C). The immunopositive signal of visfatin was observed in the cytoplasm of both cell lines, but the intensity of visfatin immunoexpression was significantly higher in BeWo cells than in JEG-3 cells, which is consistent with the results of the gene and protein expression of visfatin. Immunostaining for visfatin was also observed in commercially available normal-term placenta slides (Fig 2C); visfatin signals were found in the cytoplasm of cells, in villous cells, and blood spaces surrounding the villi.

### Gene and protein expression of visfatin in maternal and fetal parts of human term placentas

**Fig 3:**
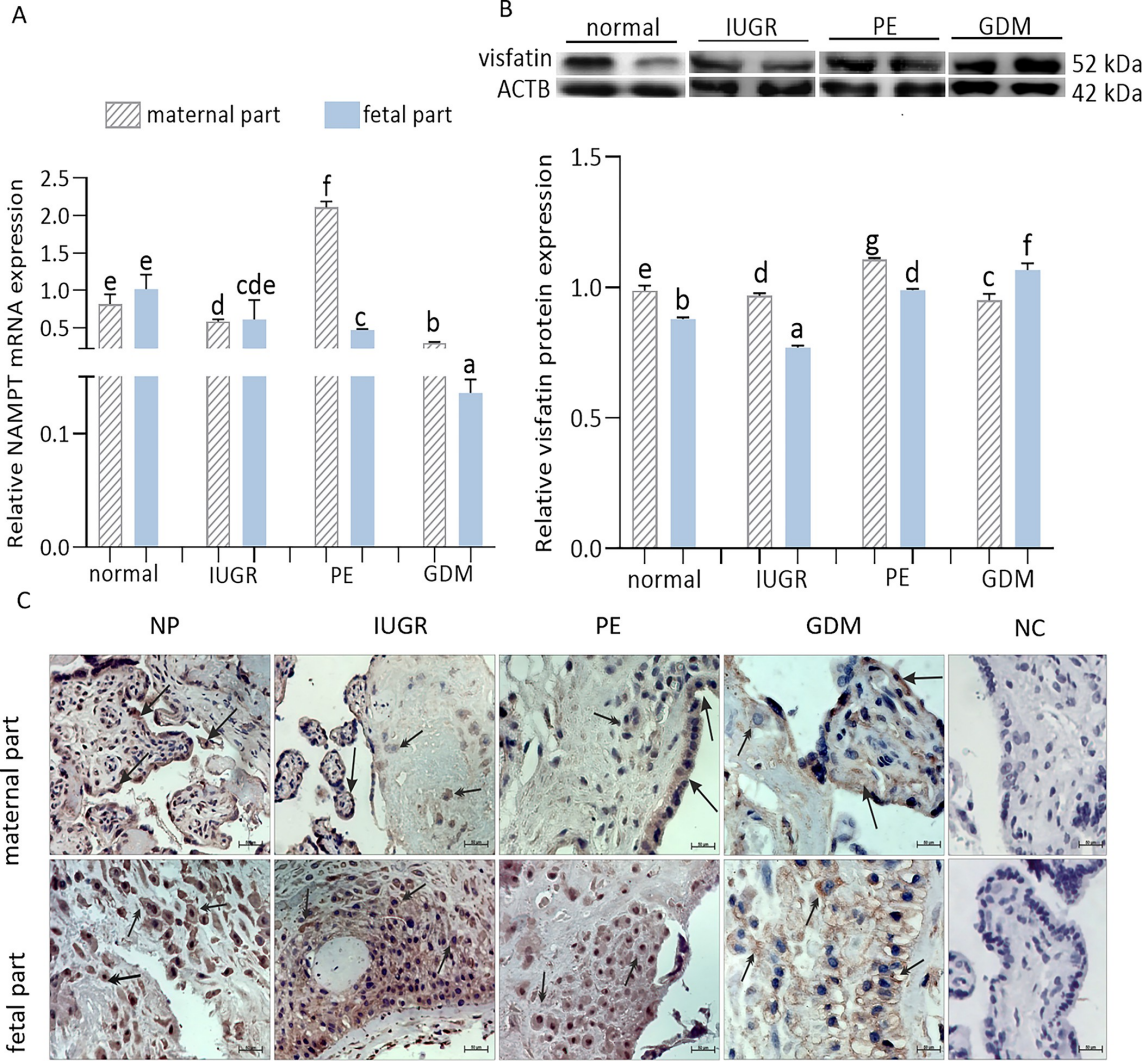
Gene (A) and protein expression (B) of visfatin (p < 0.05, mean ± SEM) as well as its immunolocalization in term placenta from normal and IUGR, PE, GDM pregnancies (C) (scale bar is 50 μm). Immunolocalization: capillary epithelium (long arrows), decidual cells (double arrowheads), syncytiotrophoblasts (double arrowheads). Fig 3B shows data from multiple blot images. The relative gene expression of visfatin was examined by qRT-PCR then the obtained results were normalized using the geometric mean of reference gene expression (GAPDH, TBP, YWHAZ) due to the comparative cycle threshold method. The protein expression of visfatin was detected by Western blot, and protein lanes were densitometrically measured and shown as the ratio relative to ACTB expression. Statistical analysis was shown using ANOVA followed by Tukey’s HSD multiple range test (mean ± SEM, p < 0.05; n = 5). NP—normal placenta; IUGR—intrauterine growth restriction; PE—preeclampsia; GDM—gestational diabetes mellitus; NC—negative control; ACTB—β-actin.

The relative NAMPT transcript levels of the maternal parts of the normal, IUGR, PE, and GDM placentas (0.82 ± 0.36, 0.58 ± 0.07, 2.11 ± 0.11, 0.29 ± 0.01) were compared with those of the fetal parts (1.02 ± 0.55, 0.61 ± 0.63, 0.47 ± 0.02, 0.14 ± 0.02), showing the highest values for the maternal part of PE placentas and the lowest values for the maternal part of GDM placentas. The highest values for the fetal part were recorded in the placentas from normal pregnancies and the lowest values for GDM placentas (Fig 3A). A significant decrease in NAMPT mRNA expression was observed in the maternal and fetal parts of IUGR, GDM placentas, and in the fetal part of PE placentas; the opposite tendency was noted for the maternal part of PE placentas. On the other hand, for relative visfatin protein expression, the highest values for the maternal part were noted in PE placentas, while the lowest values were observed in GDM placentas. For the fetal part, the highest values of visfatin were observed in GDM, and the lowest values in IUGR placentas (Fig 3B, p < 0.05). In addition, statistically significant differences were noted between the values obtained for the maternal and fetal parts of normal placentas and all placental pathologies.

Representative microphotographs show the immunolocalization of visfatin in different human placental sections (normal, PE, GDM, and IGUR) from both maternal and fetal parts (Fig 3C). In the normal maternal part of the placenta, a strong immunosignal for visfatin was present in the capillary epithelium, while in the fetal part, a strong immunosignal was observed in the syncytiotrophoblasts. Moreover, in the IGUR maternal placenta part, a strong immunosignal for visfatin was found in both the capillary epithelium and the decidual cells. On the other hand, in the IGUR fetal placenta part, the visfatin immunosignal had a high intensity in syncytiotrophoblast cells. In the maternal part of PE placentas, the visfatin immunosignal was moderate to strong in decidual cells, while in the PE fetal part, a strong visfatin immunosignal was observed in the syncytiotrophoblasts. In the GDM maternal placenta part, a moderate immunosignal for visfatin was observed in the capillary epithelium and decidual cells, while in the GDM fetal part of the placenta, a strong immunosignal for visfatin was observed in the syncytiotrophoblasts.

### Effect of P_4_ on visfatin level in JEG-3 cells and involvement of signaling pathways

**Fig 4:**
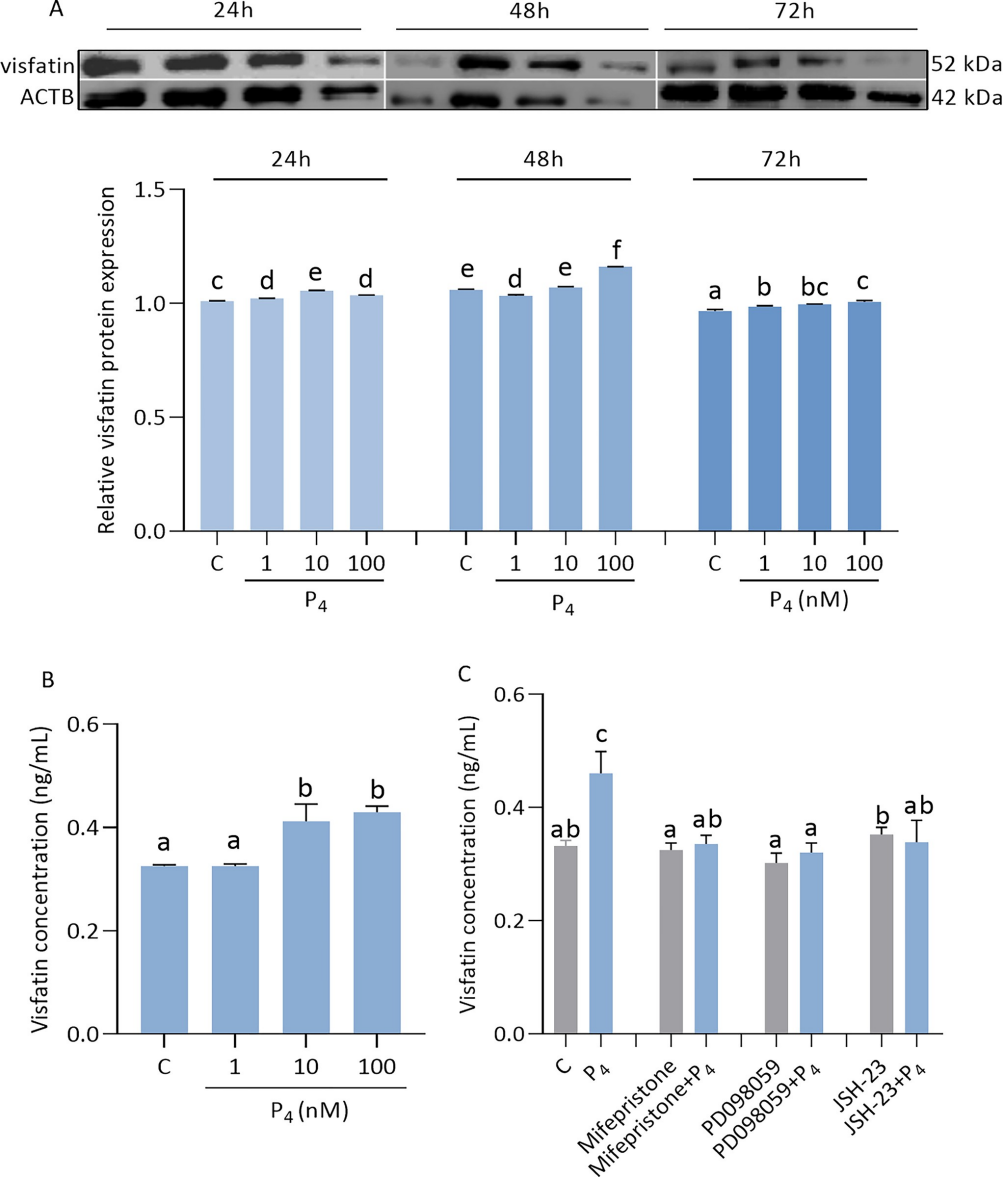
Effect of progesterone (P_4_) on visfatin levels in JEG-3 (A-C). Relative protein expression of visfatin was measured after P_4_ at doses 1, 10, 100 nM by 24, 48, 72 h (A). Visfatin concentration was measured in the culture medium after 48 h of P_4_ treatment (B). Involvement of mPR/PR, ERK1/2, and NF-κB signaling pathways in visfatin regulation by P_4_ after 48 h (C). Fig 4A shows data from multiple blot images. The protein expression of visfatin was detected by Western blot, and protein lanes were densitometrically measured and shown as the ratio relative to ACTB expression. Visfatin concentration was studied by ELISA immunoassay. Statistical analysis was shown using ANOVA followed by Tukey’s HSD multiple range test (mean ± SEM, p < 0.05; n = 3). Mifepristone—membrane (mPR)/ nuclear (PR) P_4_ receptor antagonist; PD098059—an inhibitor of the extracellular signal-regulated kinase (ERK1/2); JSH-23—an inhibitor of the transcription factor NF kappa B (NF-κB); ACTB—β-actin.

We have shown that P_4_ increased the level of visfatin in a dose- and time-dependent manner in trophoblast cells (Fig 4). We observed that P_4_ at all doses stimulated the visfatin protein expression in JEG-3 cells after 24 and 72 h of incubation. However, we noted that after 48 h, P_4_ had a modulatory effect on visfatin level: a dose of 1 nM decreased the level, a dose of 10 nM had no effect, and a dose of 100 nM increased the level (Fig 4A, p < 0.05). Interestingly, we observed that after 48 h of incubation, P_4_ at 1 nM had no effect, while at doses of 10 nM and 100 nM, P4 increased the level of visfatin in the culture medium compared with the control (0.33 ± 0.01, 0.33 ± 0.01, 0.41 ± 0.10, 0.43 ± 0.03) (Fig 4B, p < 0.05). As shown in Fig 4C, visfatin secretion was strongly reduced in cells treated with P_4_, mifepristone, PD098059, or JSH-23 (p < 0.05).

### Effect of E_2_ on visfatin level in JEG-3 cells and involvement of signaling pathways

**Fig 5:**
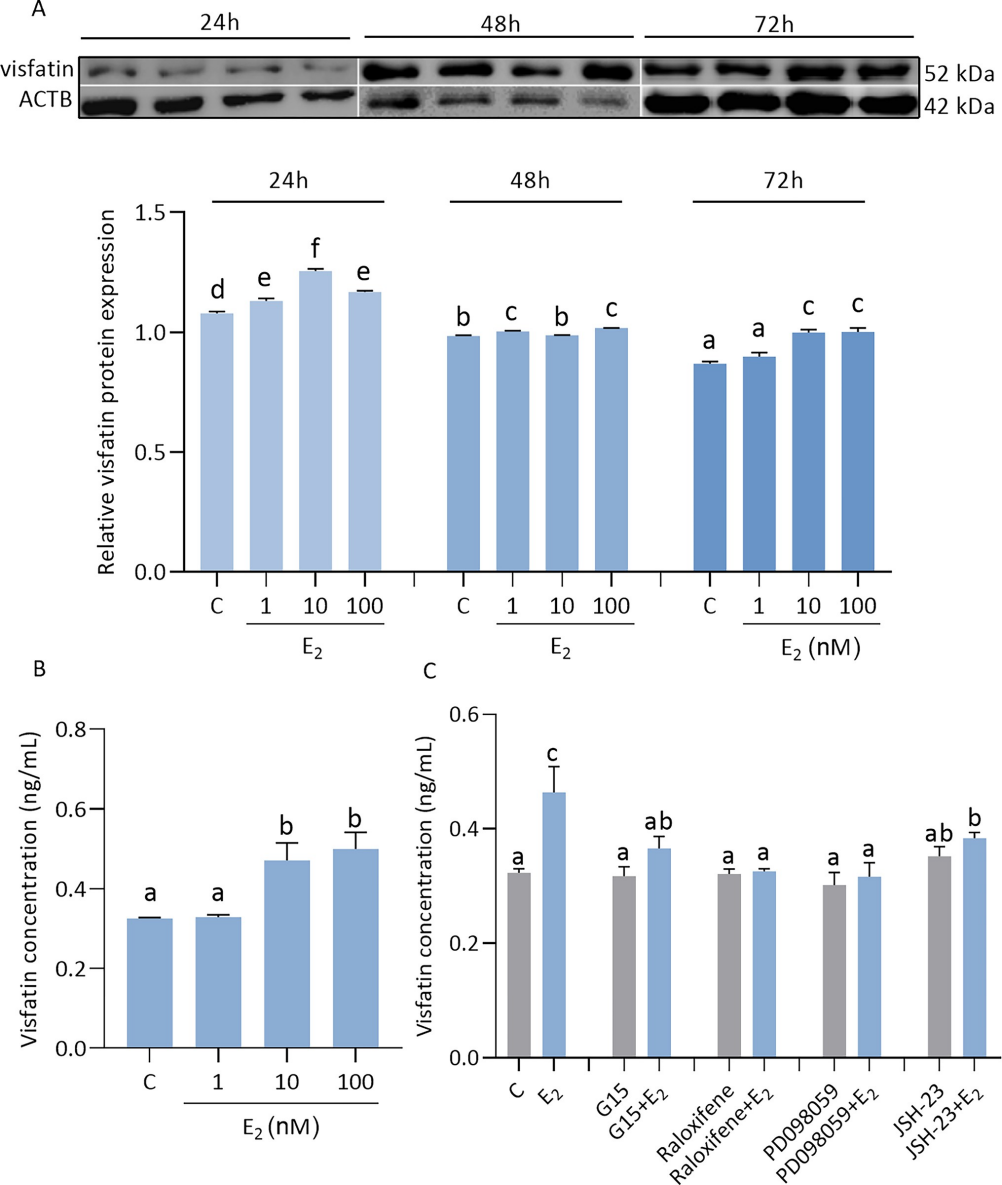
Effect of estradiol (E_2_) on visfatin levels in JEG-3 (A-C). Relative protein expression of visfatin was measured after E_2_ at doses 1, 10, 100 nM by 24, 48, 72 h (A). Visfatin concentration was measured in the culture medium after 48 h of E_2_ treatment (B). Involvement of GPR30, ER, ERK1/2, and NF-κB signaling pathways in visfatin regulation by E_2_ after 48 h (C). Fig 5A shows data from multiple blot images. The protein expression of visfatin was detected by Western blot, and protein lanes were densitometrically measured and shown as the ratio relative to ACTB expression. Visfatin concentration was studied by ELISA immunoassay. Statistical analysis was shown using ANOVA followed by Tukey’s HSD multiple range test (mean ± SEM, p < 0.05; n = 3). E2—estradiol; raloxifene–nuclear E_2_ receptor (ER) antagonist; G15 –membrane E_2_ receptor (GPR30) antagonist; PD098059—an inhibitor of the extracellular signal-regulated kinase (ERK1/2); JSH-23—an inhibitor of the transcription factor NF kappa B (NF-κB); ACTB—β-actin.

We observed a dose- and time-dependent effect of E_2_ on visfatin levels in JEG-3 cells (Fig 5). E_2_ significantly increased visfatin protein expression after 24 h at all doses, after 48 h at doses of 1 and 100 nM, and after 72 h at doses of 10 and 100 nM (Fig 5A, p < 0.05). Moreover, E_2_ stimulated visfatin level in the culture medium after 48 h at doses of 10 and 100 nM (0.33 ± 0.01, 0.33 ± 0.01, 0.47 ± 0.13, 0.50 ± 0.14) (Fig 5B, p < 0.05). We showed that visfatin concentration in the culture medium was lower in E_2_ with G15, raloxifene, PD098059, or JSH-23 treatments (Fig 5C, p < 0.05).

### Effect of hCG on visfatin level in JEG-3 cells and involvement of signaling pathways

**Fig 6:**
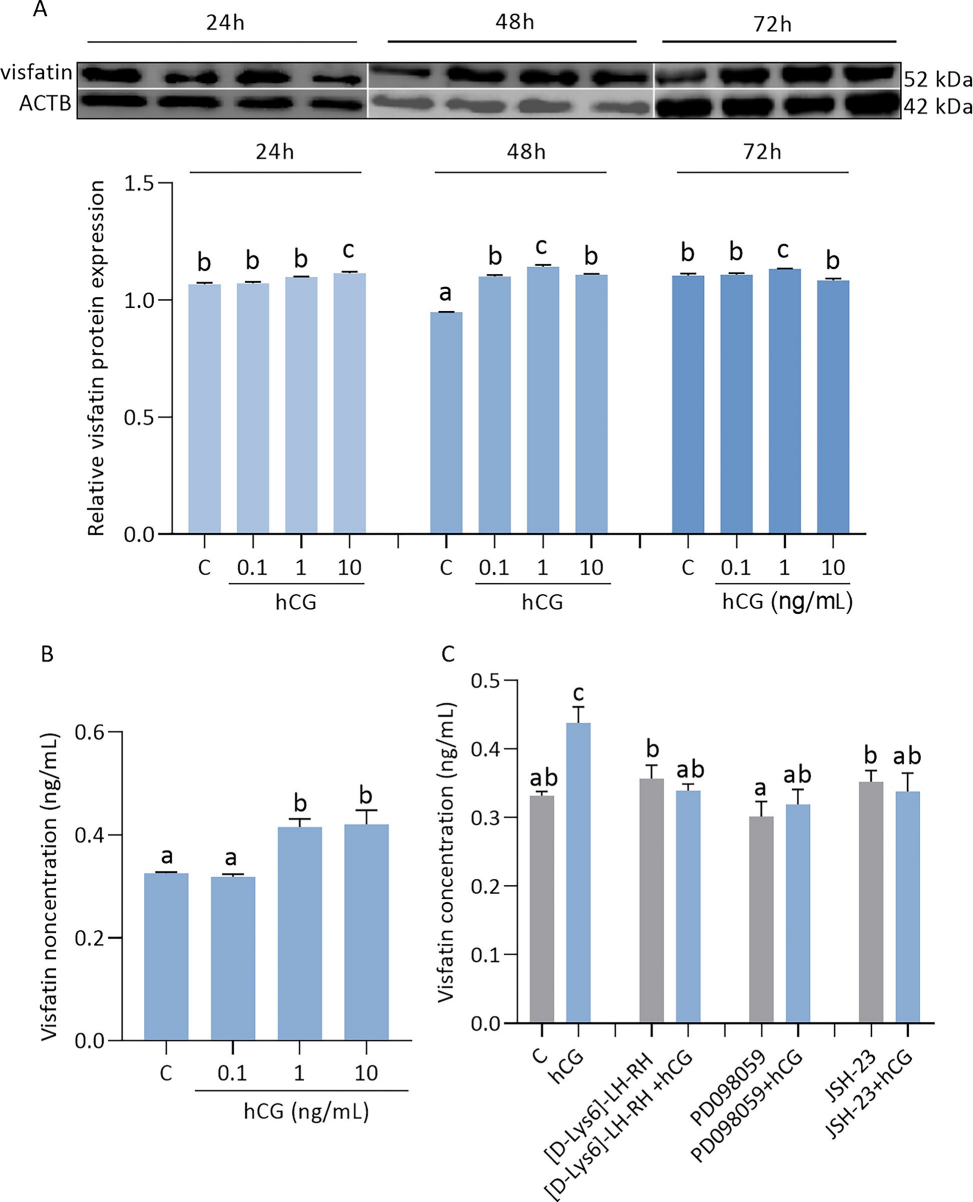
Effect of chorion gonadotropin (hCG) on visfatin levels in JEG-3 (A-C). Relative protein expression of visfatin was measured after hCG at doses 0.1, 1, 10 ng/ mL by 24, 48, 72 h (A). Visfatin concentration was measured in the culture medium after 48 h of hCG treatment (B). Involvement of LHCGH, ERK1/2, and NF-κB signaling pathways in visfatin regulation by hCG after 48 h (C). Fig 6A shows data from multiple blot images. The protein expression of visfatin was detected by Western blot, and protein lanes were densitometrically measured and shown as the ratio relative to ACTB expression. Visfatin concentration was studied by ELISA immunoassay. Statistical analysis was shown using ANOVA followed by Tukey’s HSD multiple range test (mean ± SEM, p < 0.05; n = 3). hCG—chorion gonadotropin; [D-Lys^6^]-LH-RH—hCG receptor (LHCGH) antagonist; PD098059—an inhibitor of the extracellular signal-regulated kinase (ERK1/2); JSH-23—an inhibitor of the transcription factor NF kappa B (NF-κB); ACTB—β-actin.

We found that hCG at all doses increased the protein expression of visfatin in JEG-3 cells after 48 h of incubation, as well as at a dose of 10 ng/mL after 24 h of incubation and at a dose of 1 ng/mL after 72 h of incubation (Fig 6A, p < 0.05). Also, hCG at doses of 1 and 10 ng/mL increased visfatin secretion (0.33 ± 0.01, 0.32 ± 0.02, 0.42 ± 0.05, 0.42 ± 0.07) after 48 h of incubation (Fig 6B, p < 0.05). As shown in Fig 6B, visfatin levels in the culture medium were decreased by hCG with [D-Lys6]-LH-RH, PD098059, or JSH-23 (p < 0.05).

### Effect of INS on visfatin level in JEG-3 cells and involvement of signaling pathways

**Fig 7:**
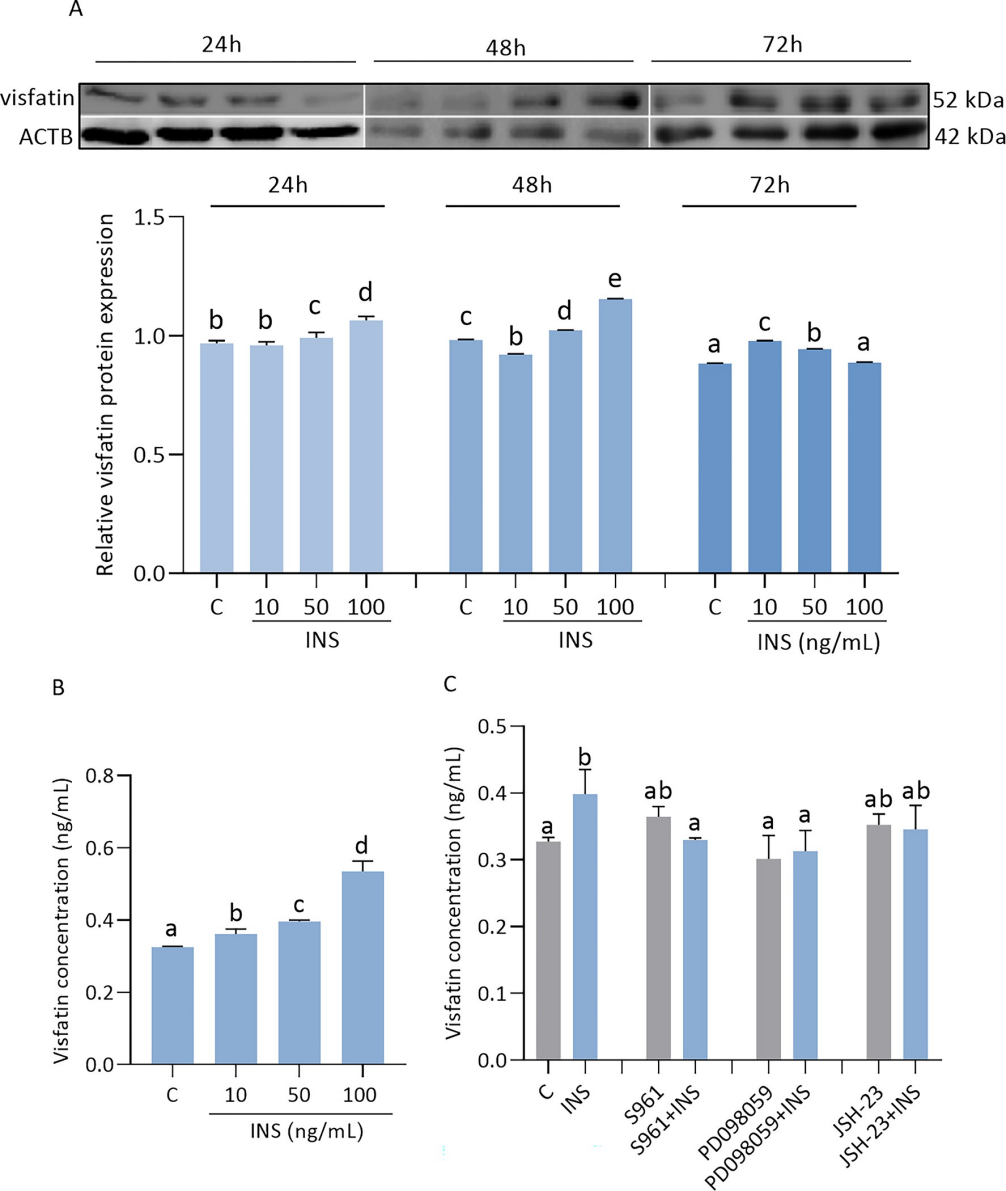
Effect of insulin (INS) on visfatin levels in JEG-3 (A-C). Relative protein expression of visfatin was measured after INS at doses 10, 50, 100 ng/ mL by 24, 48, 72 h (A). Visfatin concentration was measured in the culture medium after 48 h of INS treatment (B). Involvement of INSR, ERK1/2, and NF-κB signaling pathways in visfatin regulation by INS after 48 h (C). Fig 7A shows data from multiple blot images. The protein expression of visfatin was detected by Western blot, and protein lanes were densitometrically measured and shown as the ratio relative to ACTB expression. Visfatin concentration was studied by ELISA immunoassay. Statistical analysis was shown using ANOVA followed by Tukey’s HSD multiple range test (mean ± SEM, p < 0.05; n = 3). INS—insulin; S961 –INS receptor (INSR) antagonist; PD098059—an inhibitor of the extracellular signal-regulated kinase (ERK1/2); JSH-23—an inhibitor of the transcription factor NF kappa B (NF-κB); ACTB—β-actin.

The results revealed that INS increased visfatin protein expression in JEG-3 cells after 24 h at doses of 50 and 100 ng/ mL, as well as after 72 h at doses of 10 and 50 ng/mL (Fig 7A, p < 0.05). We noted that INS after 48 h of incubation at a dose of 10 ng/mL decreased visfatin protein expression, while INS at doses of 50 and 100 ng/mL had a stimulatory effect (Fig 7A, p < 0.05). Moreover, INS at all doses significantly increased the visfatin level in the cultured medium after 48 h (0.33 ± 0.01, 0.36 ± 0.05, 0.40 ± 0.01, 0.53 ± 0.09) (Fig 7B, p < 0.05). We demonstrated that INS with S961 or PD098059 decreased visfatin levels in the culture medium (Fig 7C, p < 0.05).

## Discussion

In our study, we demonstrated for the first time the expression and immunolocalization of visfatin in human trophoblast JEG-3 and BeWo cells as well as in terminal placentas from normal pregnancies and pathologies such as IUGR, PE, and GDM. Furthermore, we also revealed a dose- and time-dependent regulation of visfatin levels by E_2_, P_4_, hCG, and INS, along with the involvement of different molecular signaling pathways, including mPR/PR, GPR30, ER, LHCGR, INSR, ERK1/2, and NF-κB, in visfatin regulation.

We demonstrated that mRNA and protein expression of visfatin increased in *in vitro* cultures of JEG-3 and BeWo placental cells after 24, 48, and 72 h. Also, a significantly higher expression of the visfatin gene and protein was observed in BeWo cells than in JEG-3 cells. Our results are in good agreement with data obtained by Ma et al. [32] and Zhang et al. [33], who observed visfatin transcript and protein in BeWo cells. Moreover, our data indicated the cytoplasmic localization of visfatin in placental cell lines, with a stronger signal in BeWo cells than in JEG-3 cells. BeWo cells possess characteristics of the syncytiotrophoblast and extravillous trophoblast and serve as an *in vitro* model for investigating trophoblast fusion [34]. Also, BeWo cells are commonly used to study syncytialization, adhesion, and endocrine function, while JEG-3 lines are widely used to study the molecular mechanisms underlying the proliferation and invasive potential of cytotrophoblasts [23, 35]; this particular localization suggests a direct role of visfatin in the function of human placenta cells. To confirm this hypothesis, other adipokines, such as apelin, leptin, or adiponectin, were investigated as a regulator of placenta hormone secretion, proliferation, apoptosis, survival, and invasion [18].

Interestingly, the obtained results compared visfatin expression in the maternal and fetal parts of placentas from healthy women and women with pregnancy pathology. During pregnancy, the placenta (a transitional fetal organ) is formed 1 week after fertilization and is created by the cyto- and syncytiotrophoblast layers. The placenta plays an important role in the proper development of the fetus: it creates a placental barrier, transports substances between the mother and the child, and produces and secretes hormones [18]. However, in pregnancy pathologies, the placenta is altered in many ways. For example, in PE, the placental tissue is characterized by a disordered structure and cavities [36]. In GDM, the placenta has intervillous edema with increased content of fibrinous material, as well as sporadic areas of calcification or fibrinous necrosis [37]. In IUGR, there is an increase in syncytial knots and villous vascular structures [38]. The results of our study showed for the first time the expression of the visfatin transcript and protein decreased in the IUGR placenta compared with the placenta of a normal pregnancy. Literature data have shown contradictory results (decreased, increased, no difference) of visfatin expression in placental cells of GDM versus normal placentas [32, 39, 40]. In our studies, we observed decreased visfatin mRNA in the maternal and fetal parts of GDM placentas compared with normal placentas, while increased protein levels were found in the fetal parts. Previous studies on embryonic stem cells suggest that differences in mRNA and protein levels are due to translational and post-translational regulation [41]. Moreover, Schwanhäusser et al. [42] showed that mRNA levels explain about 40% of the variability in protein levels; most of this variation is a consequence of differences in transcription rates, while mRNA stability plays a minor role. Furthermore, the obtained results showed that mRNA and protein expression of visfatin was the highest in the maternal part of the PE placentas, while transcript levels were decreased in the fetal part of the PE placentas. The highest expression of visfatin mRNA and protein in the maternal part of PE placentas might indicate a potential role for visfatin as a new marker in the diagnosis of pregnancy disorders. The expression and concentration of visfatin were measured in lean, term pregnant, obese, and diabetic obese women, and the results showed an increase in visfatin mRNA in pregnant women compared with all tested groups [6]. Visfatin serum concentrations in pregnant women were estimated to be approximately 40 ng/mL, which corresponds to previous reports indicating a level of 18.83 ± 4.27 ng/mL [6, 15]. Moreover, subsequent studies show a higher concentration of visfatin in the plasma of PE women (63.8 ± 4.9 ng/mL) compared with women in the normal pregnancy group (43.6 ± 7.8 ng/mL) and the non-pregnant control group (31.6 ± 4.2 ng/mL) [43]. Also, the level of circulating visfatin is significantly lower in the fetus than in the mother during normal, PE, or IUGR pregnancies, which is in agreement with our results on the differences in visfatin protein expression between the maternal and fetal parts of the mentioned disorders. Unfortunately, this relationship is not fully explained, although the greater fat storage in the mother’s body in comparison with the newborn may play an important role [17]. However, one of the earlier studies, conducted only on the material from normal pregnancies, contrasts with this report, indicating no significant differences in the levels of visfatin between mothers and newborns, although the main reason for the observed discrepancies may be ethnicity, gestational age, or clinical diagnosis at the time of inclusion for research [15].

Our results confirmed studies on the immunolocalization of visfatin protein in the maternal and fetal parts of terminal placentas from normal and pathological pregnancies. Morgan et al. [6] showed that visfatin protein is translated in the human placenta, particularly in syncytiotrophoblasts and fetal capillary endothelium. Also, higher visfatin staining was observed in the syncytiotrophoblasts of obese women than in those of lean women, indicating a potential role for visfatin as a marker of obesity-related pregnancy complications [44]. The obtained results have shown a strong visfatin signal in the syncytiotrophoblasts of the fetal parts of the placentas in all study groups, while in the maternal part, we observed visfatin staining in the capillary epithelium and, additionally, in decidual cells for each of the pathologies (as opposed to normal placentas). The decidual cells are the main type of resident cell at the fetal-maternal interface, accounting for 40% of all cells [45]. They arise from endometrial stromal cells in response to P_4_ during the "implantation window" of the secretory phase during the menstrual cycle and occur throughout pregnancy. Moreover, they play an important role in blastocyst implantation, trophoblast invasion, maternal-fetal immune responses, and decidualization through autocrine and paracrine interactions [46]. It has been shown that decidual cell dysfunction may be one of the reasons for the development of PE, which in turn often leads to the development of IUGR. The abnormal infiltration of immune cells seen in PE may be due to the dysregulated production of chemoattractants by decidual cells [47]. PE is associated with decidual hemorrhage, in which excessive thrombin formation by binding of decidual cell-derived tissue factor to circulating factor VIIa leads to increased production of anti-angiogenic sFlt-1 [48]. Moreover, Birdir et al. [49] found a link between IUGR and lower levels of placental growth factor (PLGF) and visfatin, which may be an indicator of IUGR. These data, as well as reports obtained from other cells, such as umbilical cord or endothelial cells [50, 51], indicated the pro-angiogenic properties of visfatin, suggesting that it might have a protective effect on PE development; however, the role of visfatin in the decidual cells of PE or IUGR placentas requires clarification in further research.

The results of the current study indicate that P_4_, E_2_, hCG, and INS regulate the level of visfatin in human placental cells. Previous studies have shown that maternal plasma visfatin levels vary between 11–14, 19–26, and 27–34 weeks of gestation, with the highest median values observed in the 2^nd^ trimester [17]. Can changes in the levels of P_4_, E_2_, hCG, and INS be the direct cause of fluctuating visfatin levels during pregnancy? As we know, the levels of P_4_, E_2_, or INS increase with the course of pregnancy, and its fastest increase is observed between the 2^nd^ and the 3^rd^ trimester, which, as was mentioned above, roughly coincides with the observed increased level of visfatin in the 2^nd^ trimester. In a normal pregnancy, the highest concentrations of P_4_ and E_2_ in maternal plasma are observed just before delivery [18]. Serum visfatin levels increase during pregnancy, and in pathologies such as IUGR, PE, and GDM, these values are even higher [15–17]. Previous studies showed that co-administration of dexamethasone and P_4_ prevented reductions in rat fetal and placental weight, as well as placental GLUT expression, indicating that P_4_ prevents IUGR [52]. Our studies have shown that P_4_ increased the level of visfatin in placental cells, which can be a side effect of the hormone or a compensatory mechanism of the endocrine machinery, helping to identify or combat pathology or, on the contrary, to aggravate the pathological condition. However, further studies are needed to clarify the relationship between P_4_ and visfatin. Moreover, dysregulated placental and uterine remodeling have been shown to result in the upregulation of E_2_ and its signaling pathway in an animal model of gestational diabetes to maintain pregnancy [53]. We demonstrated that E_2_ increased the concentration and expression of visfatin in an *in vitro* culture of placental cells, which, as mentioned above, may have a threefold nature. During PE pregnancy, the level of hCG is elevated compared with the normal control, so it is a significant indicator of pathology in the early 2^nd^ trimester of pregnancy and has some predictive value during the 1^st^ trimester of pregnancy [54]. Moreover, in the 1^st^ trimester of pregnancy, low hCG levels significantly increased the risk of IUGR, preterm delivery, low birth weight, and low Apgar scores, while high hCG levels significantly reduced the risk of preterm delivery and GDM. However, in the 2^nd^ trimester of pregnancy, both in the group with low and in the group with high concentrations of hCG, a significantly increased risk of spontaneous abortion, IUGR, and preterm delivery was found [55]. Thus, results showing increased serum visfatin levels during pregnancies complicated by GDM or IUGR [15, 17], as well as our results indicating that hCG increased visfatin levels in placenta cells, lead to a potential role of visfatin in the prediction and compensation or generation of symptoms of pregnancy disorders. Insulin sensitivity decreases by about two-thirds in both pregnant women with normal glucose tolerance (NGT) and pregnant women with GDM. Fortunately, in NGT pregnancy and most cases of GDM, insulin sensitivity is restored after delivery, although about one-third of women with GDM have reduced insulin sensitivity [56]. We observed that insulin increased the level of visfatin, which in relation to its insulin-mimetic function may suggest a protective role in the case of insulin sensitivity during pregnancy, but further research is needed to explain the suggested relations. The common denominator of this part of the research is the observed increase in the level and expression of visfatin in response to the action of placental hormones, which suggests that the level of adipokines may be regulated by the hormonal balance at the different stages of pregnancy. In the future, this knowledge may help in earlier identification of certain pregnancy pathologies and in reducing the risk of developing these pathologies. In order to better understand the mechanism of regulation of visfatin levels in the placenta, we decided to study the potential signaling pathways involved in this process. The expression and levels of many adipokines are regulated by placental hormones. E_2_, hCG, and INS increase the level of leptin in the placenta, while P_4_ reduces this level [18]. Both E_2_ and P_4_ decrease the secretion of resistin, while INS increases it [57]. In our studies, we observed an increase in visfatin levels after the action of all placental hormones; however, previous studies conducted on reproductive tissues show that E_2_ increases and P_4_ decreases the expression of visfatin in the uterus of mice during the oestrus cycle [58], while hCG increases the concentration of adipokines in the follicular fluid of women undergoing controlled ovarian stimulation [59]. Taken together, these data may indicate tissue-dependent effects of the tested hormones on adipokine levels. Moreover, previous research suggests that the ERK1/2 and NF-κB signaling pathways may be involved in the mechanisms of adipokine level regulation by placental hormones, which we also confirmed in our study; NF-κB was an exception only for INS [60, 61]. It is also worth noting that research on the regulation of adipokine levels (mainly leptin and adiponectin) indicates the potential involvement of membrane receptors or membrane and nuclear receptors of the aforementioned placental hormones; the involvement of receptors was studied by using their respective antagonists, for example, mifepristone (membrane and nuclear receptor of P_4_), raloxifene (nuclear E_2_ receptor), or S961 (INSR) [62–64]. In addition, in the available literature, we also found an antagonist of G15 (membrane E_2_ receptor) and [D-Lys6]-LH-RH (LHCGR receptor) [28, 29], which, like the previously mentioned substances, effectively blocked the tested signaling pathways, confirming the participation of all tested hormones in the regulation of visfatin levels in human placental cells.

## Conclusion

In this study, we for the first time investigated both the mRNA and protein expression of visfatin in JEG-3 and BeWo cells in the maternal and fetal parts of normal and pathological placentas. The immunolocalization of visfatin was examined in the cytoplasm of both cell lines, the syncytiotrophoblasts of the placental fetal part, and the capillary epithelium of the maternal part; in the pathologies, immunolocalization of visfatin was also examined in decidual cells. Moreover, all tested hormones increase the visfatin level in JEG-3 cells with the involvement of specific signaling pathways. Differences in the level of visfatin expression between cell lines may indicate a potentially different effect or significance of adipokines in the different layers of placental cells: extracellular and villous cytotrophoblasts. Moreover, changes in the expression and localization of visfatin in the maternal and fetal compartments of normal and pathological placentas suggested that visfatin may be a potential marker for the diagnosis of pregnancy disorders. In addition, a variable level of visfatin during individual trimesters may be the result of pregnancy hormones, which stimulate visfatin levels in human placenta cells. In addition, to confirm the obtained results, we plan future experiments on the effect of visfatin on placenta cell function.

## Supporting information

S1 Raw images (PDF)

S2 Raw images (PDF)
