# Supplementary material for: Visfatin (NAMPT) expression in human placenta cells in normal and pathological conditions and its hormonal regulation in trophoblast JEG-3 cells
Source: PLoS One. 2024 Sep 18;19(9):e0310389. doi: 10.1371/journal.pone.0310389 (PMC11410215; doi:10.1371/journal.pone.0310389)
Supplement: S1 Raw images — (PDF) [file pone.0310389.s001.pdf]

Representative original blots to protein expression of visfatin and ACTB in the JEG-3 and BeWo cells (Fig. 2B).

JEG-3

VISFATIN: 52 kDa

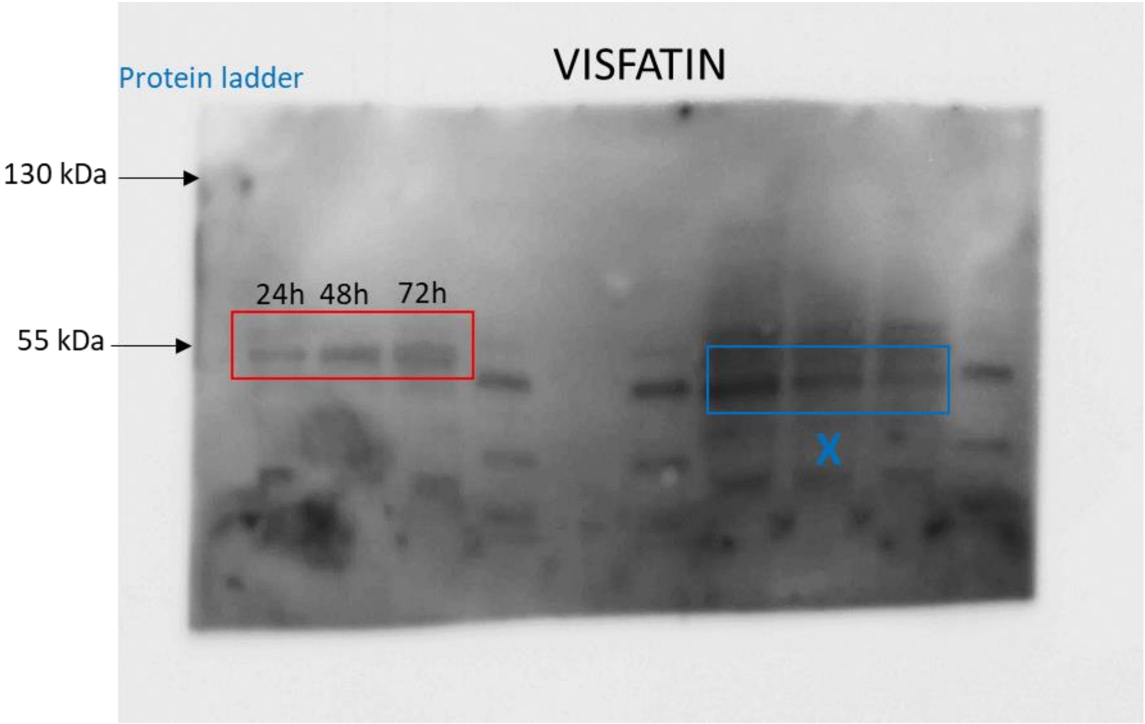

ACTB: 42kDa

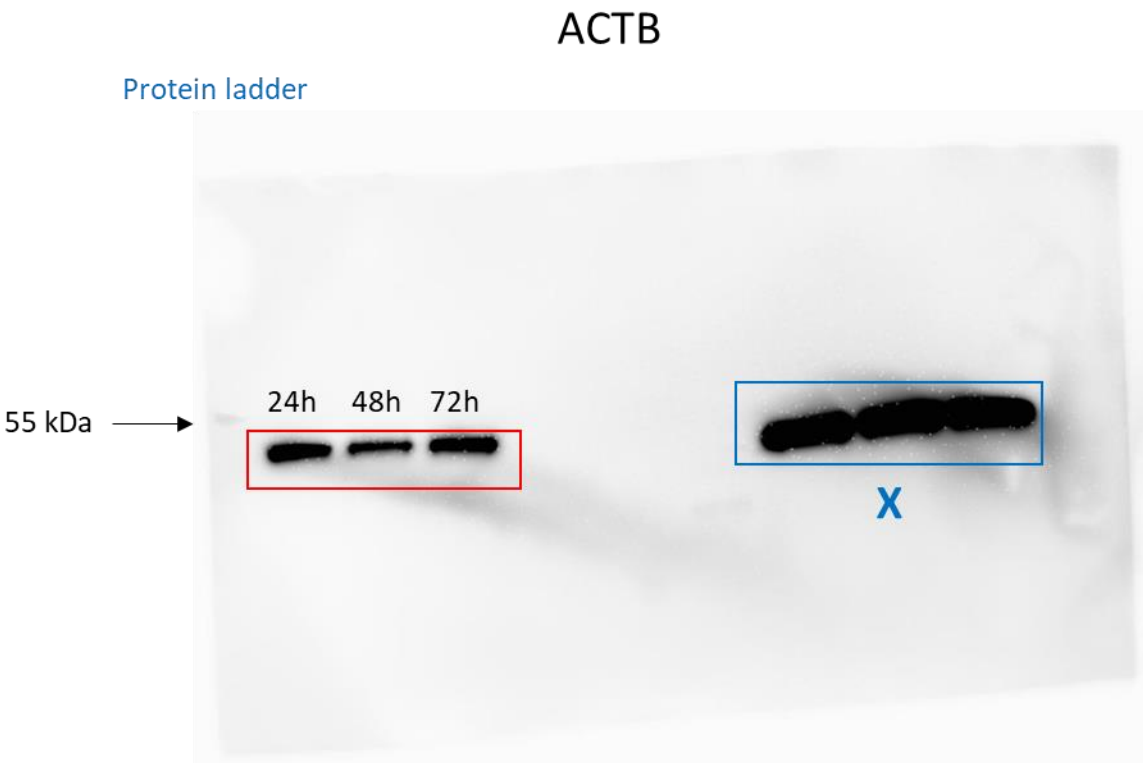

BeWo:

VISFATIN: 52 kDa

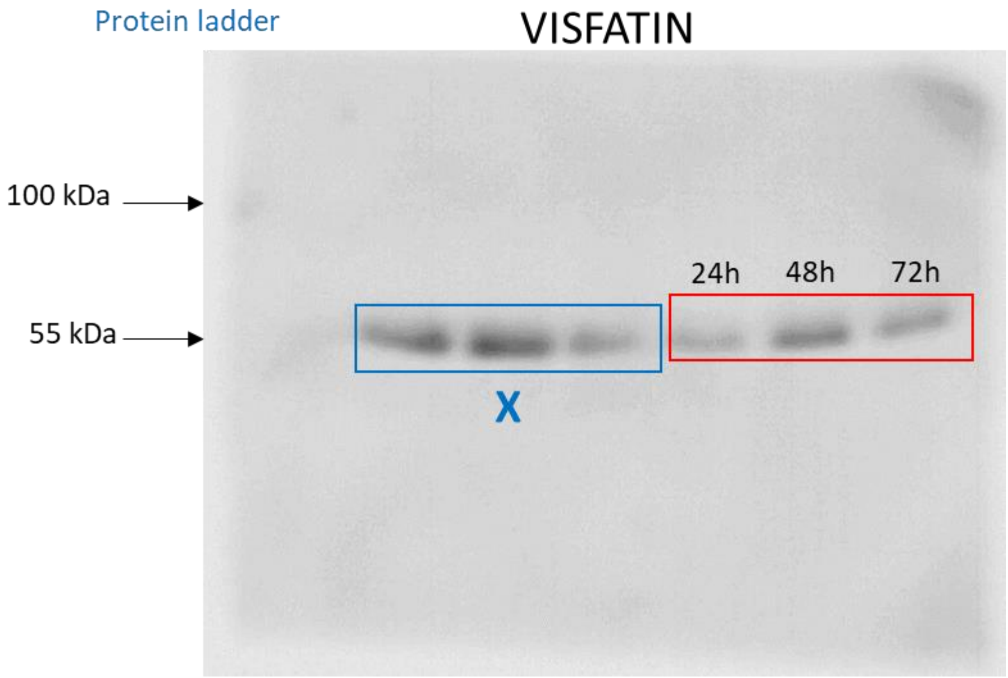

ACTB: 42kDa

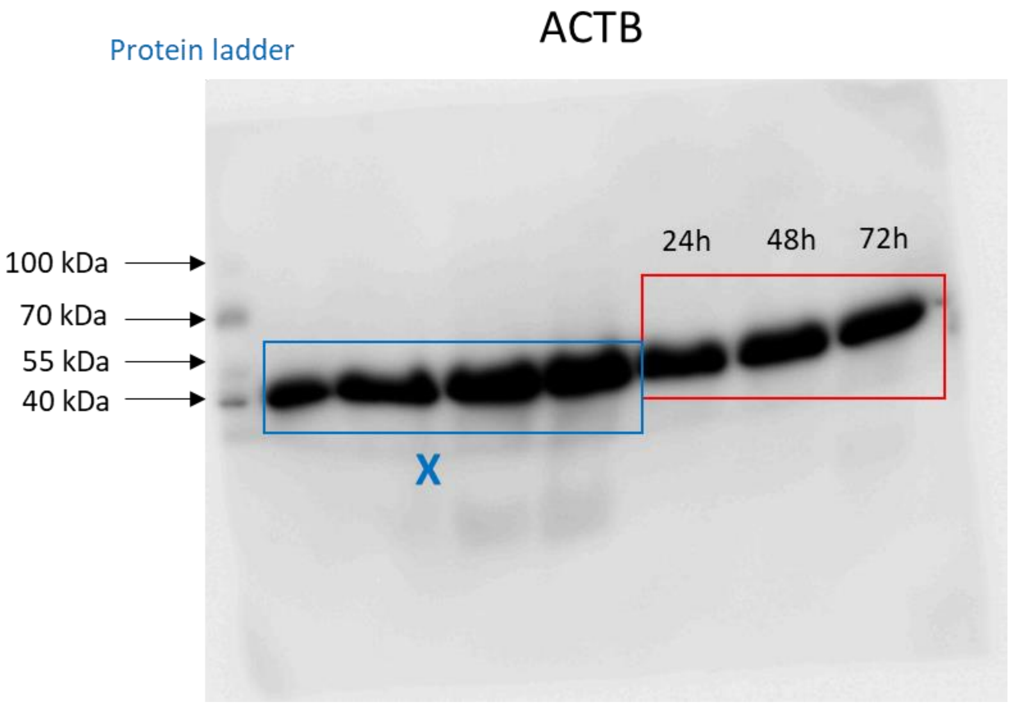

Representative original blots to protein expression of visfatin and ACTB in the normal, IUGR, PE, GDM placentas (Fig. 3B).

NORMAL PLACENTA

VISFATIN: 52 kDa

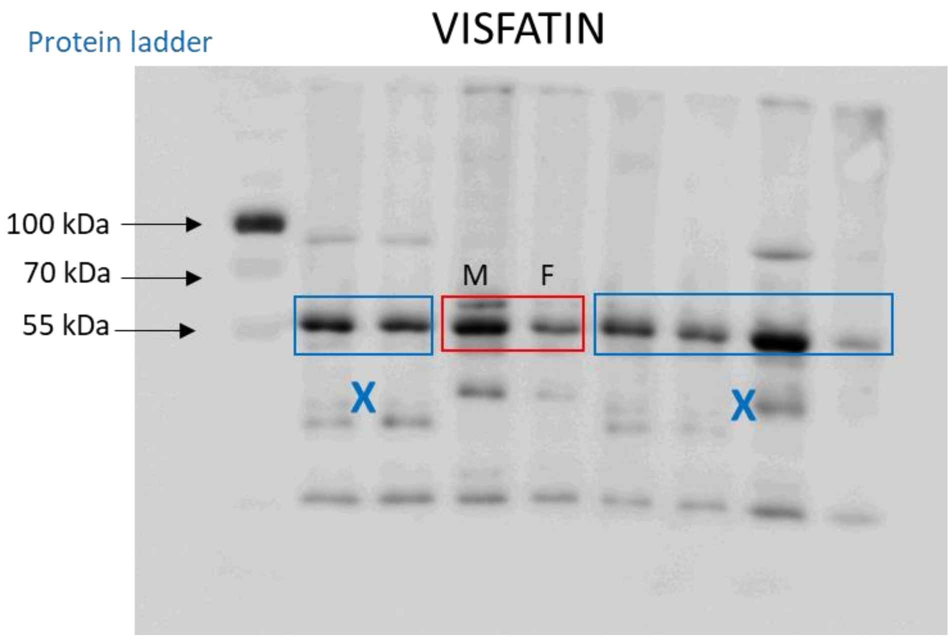

ACTB: 42kDa

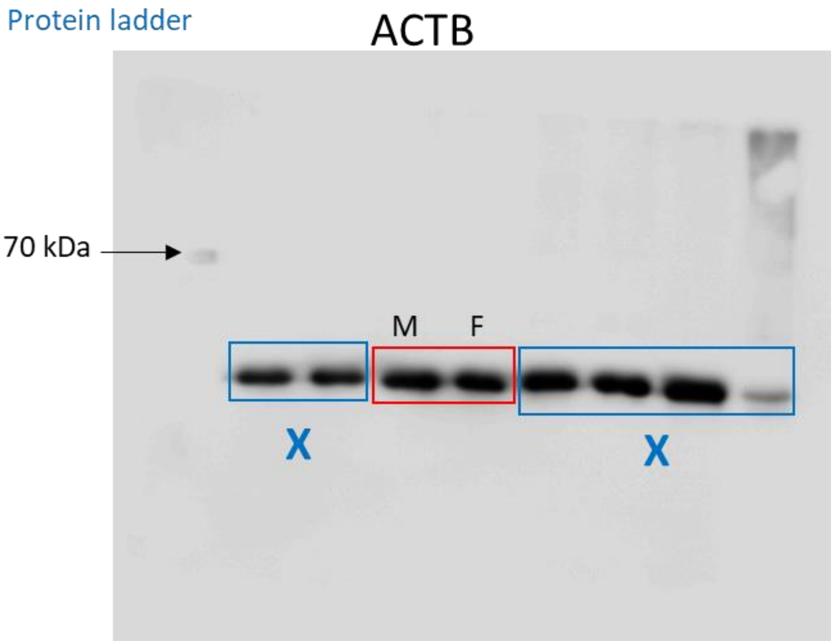

IUGR PLACENTA

VISFATIN: 52 kDa

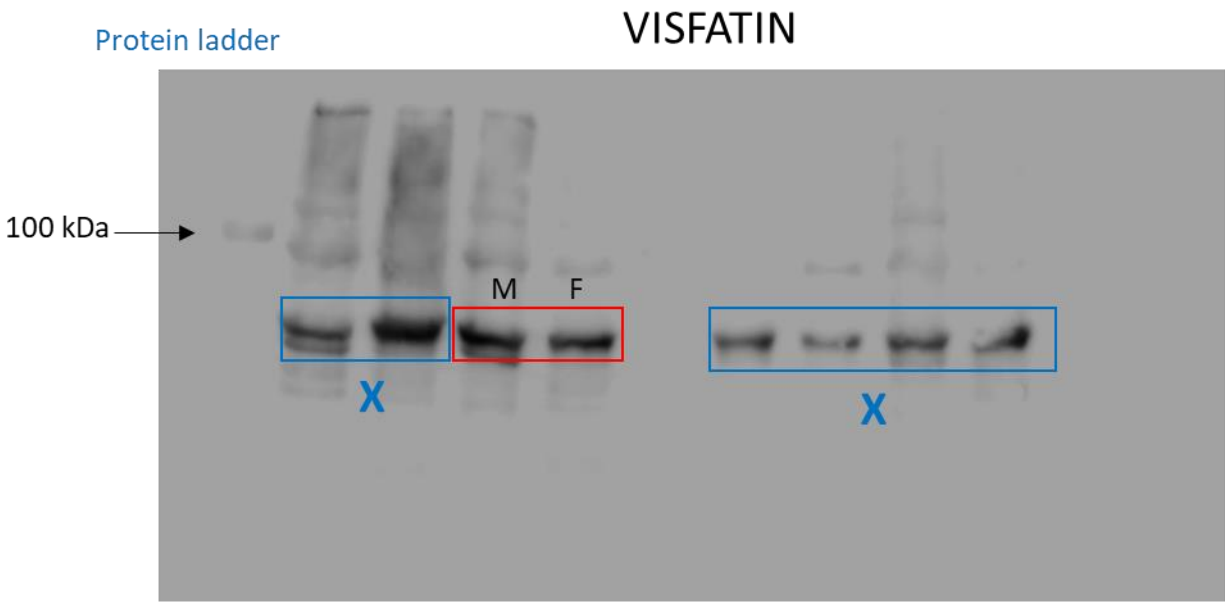

ACTB: 42kDa

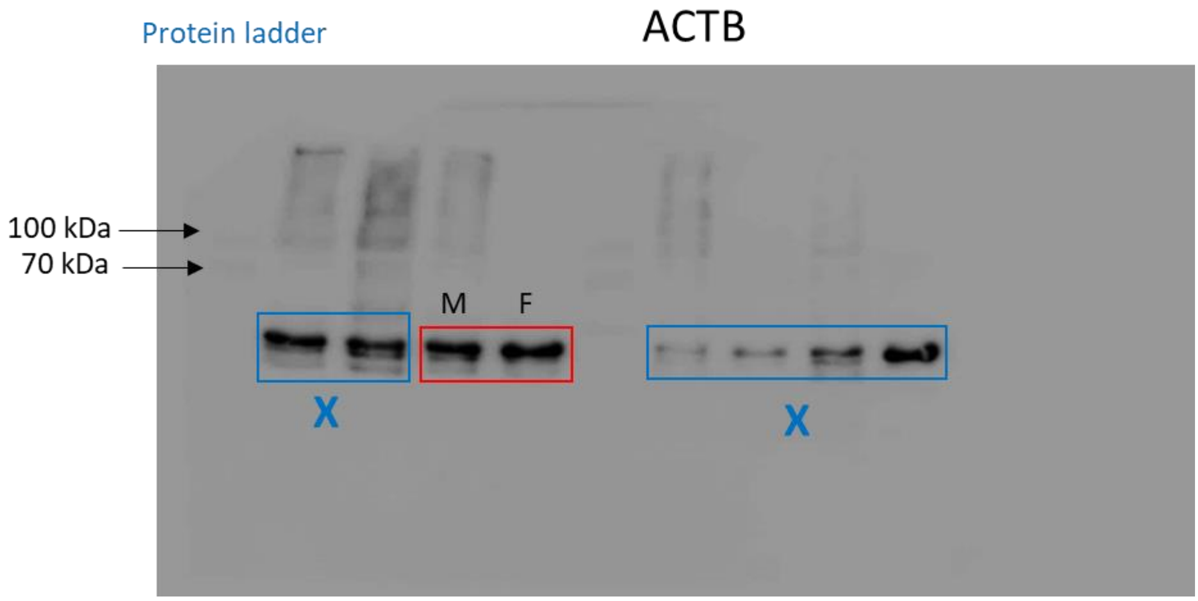

PE PLACENTA

VISFATIN: 52 kDa

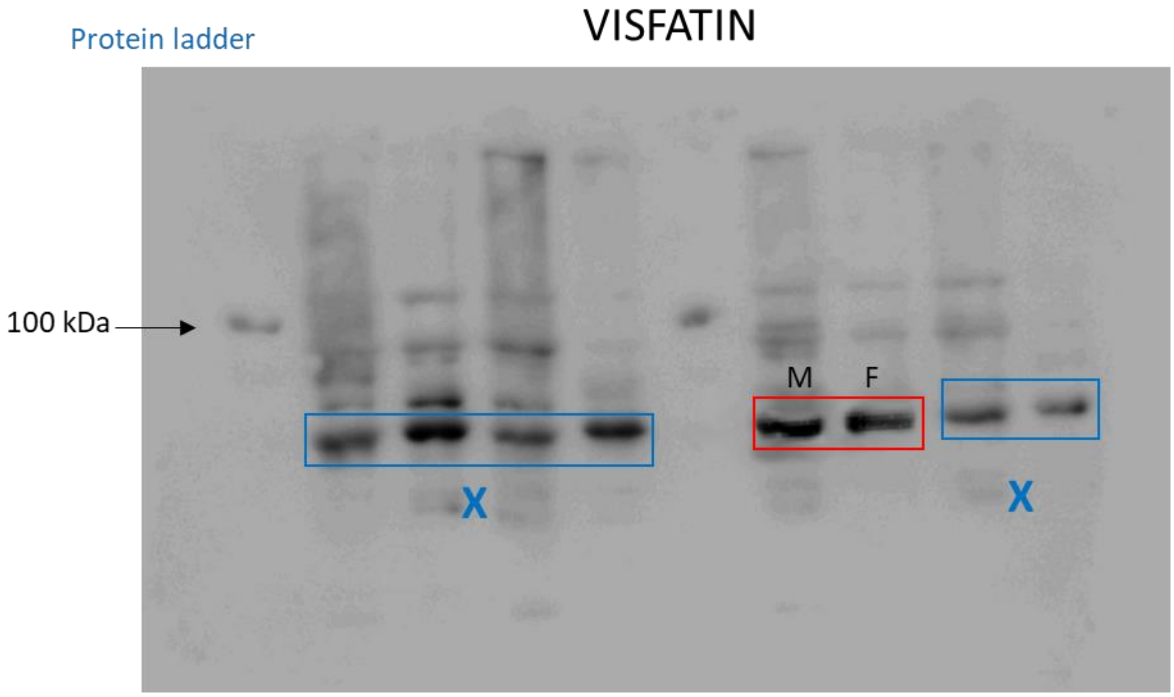

ACTB: 42kDa

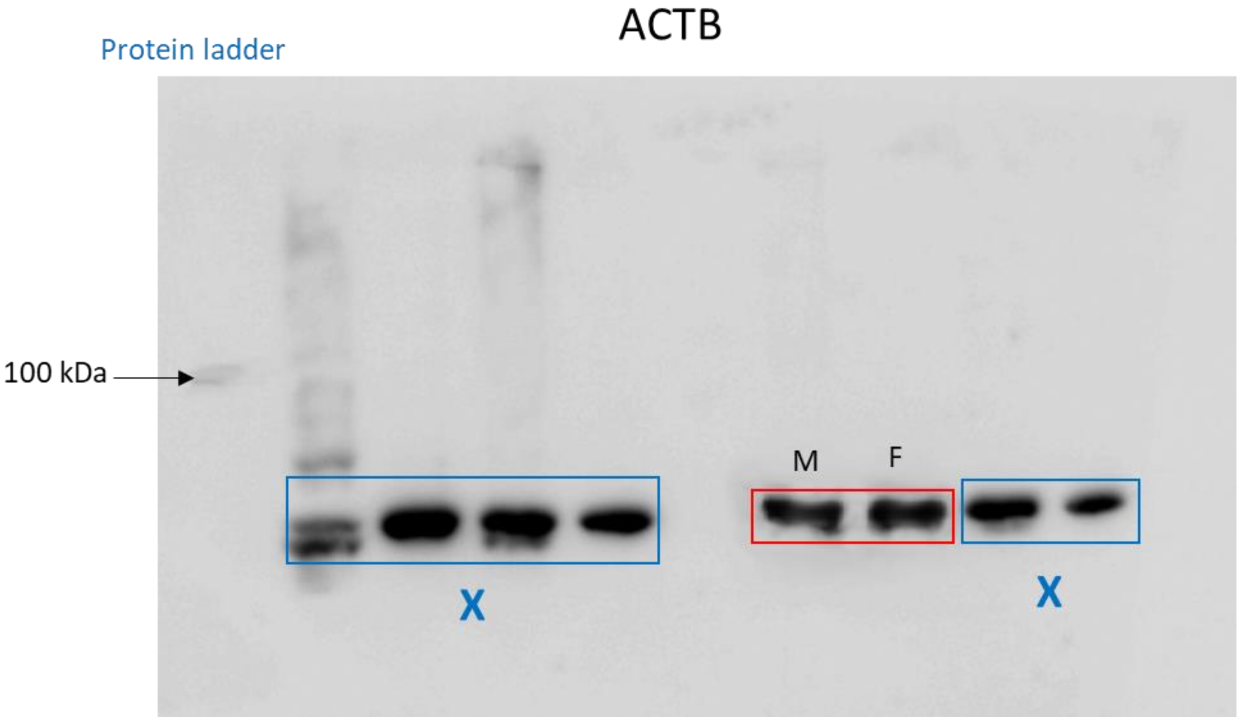

GDM PLACENTA

VISFATIN: 52 kDa

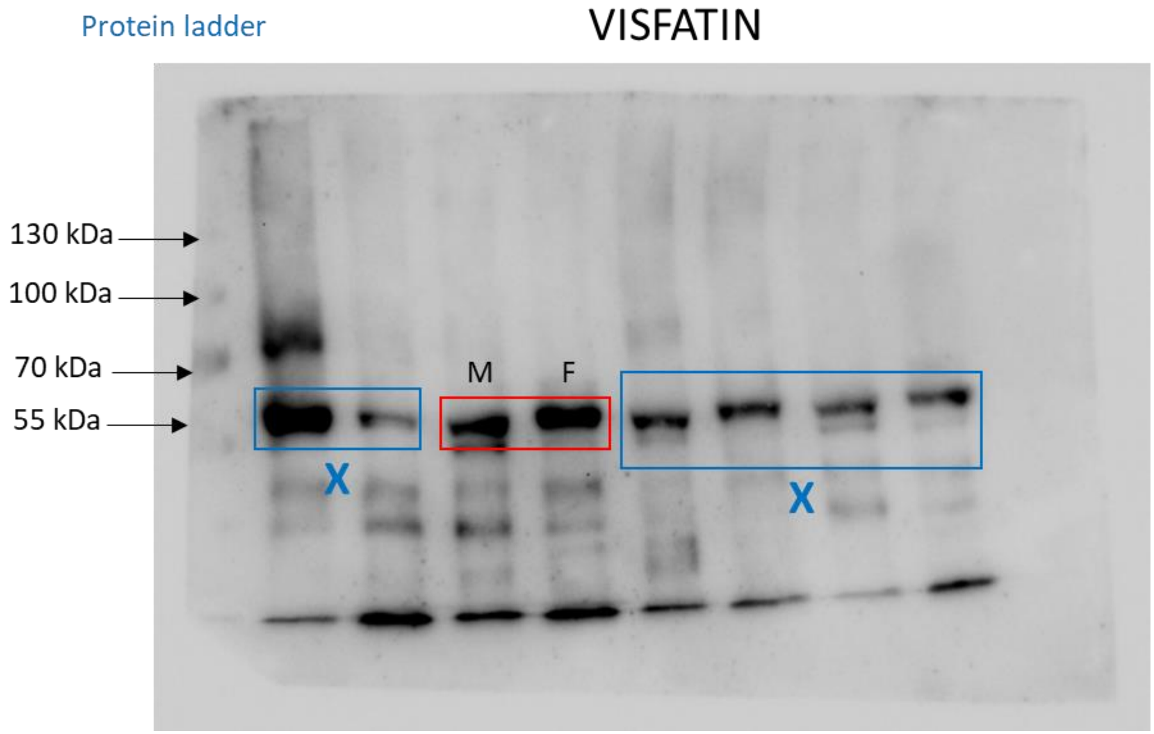

ACTB: 42kDa

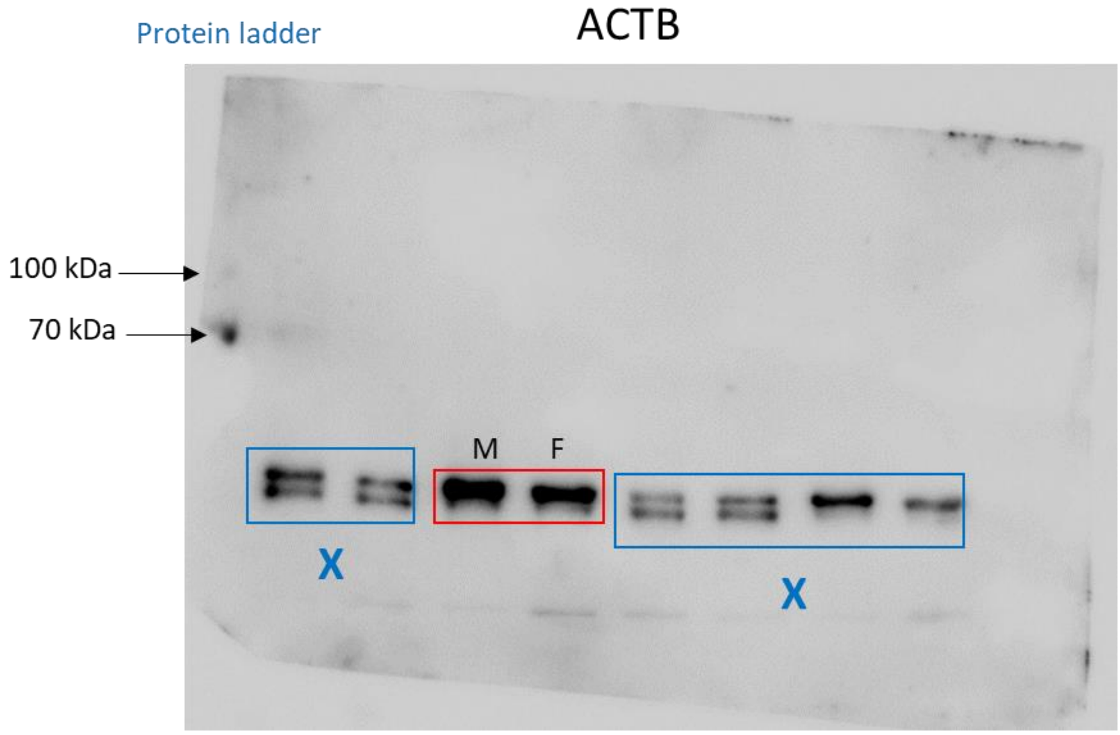

Representative original blots to regulation of visfatin protein expression by P_4 and ACTB JEG-3 (Fig. 4A).

24h:

VISFATIN: 52 kDa

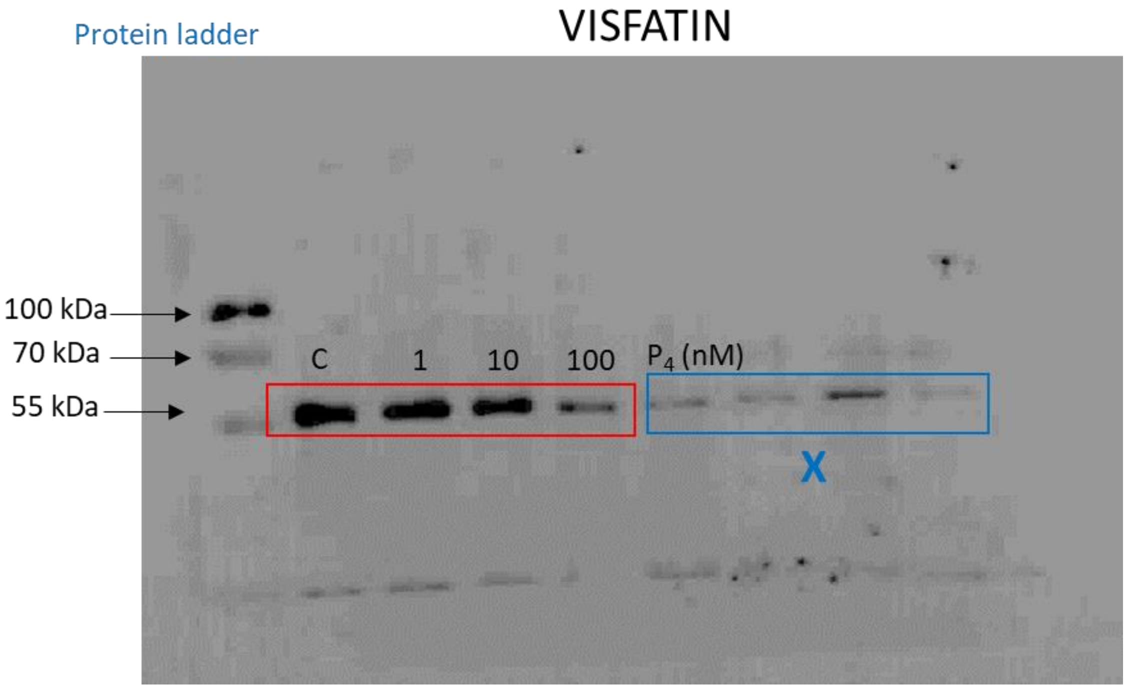

ACTB: 42kDa

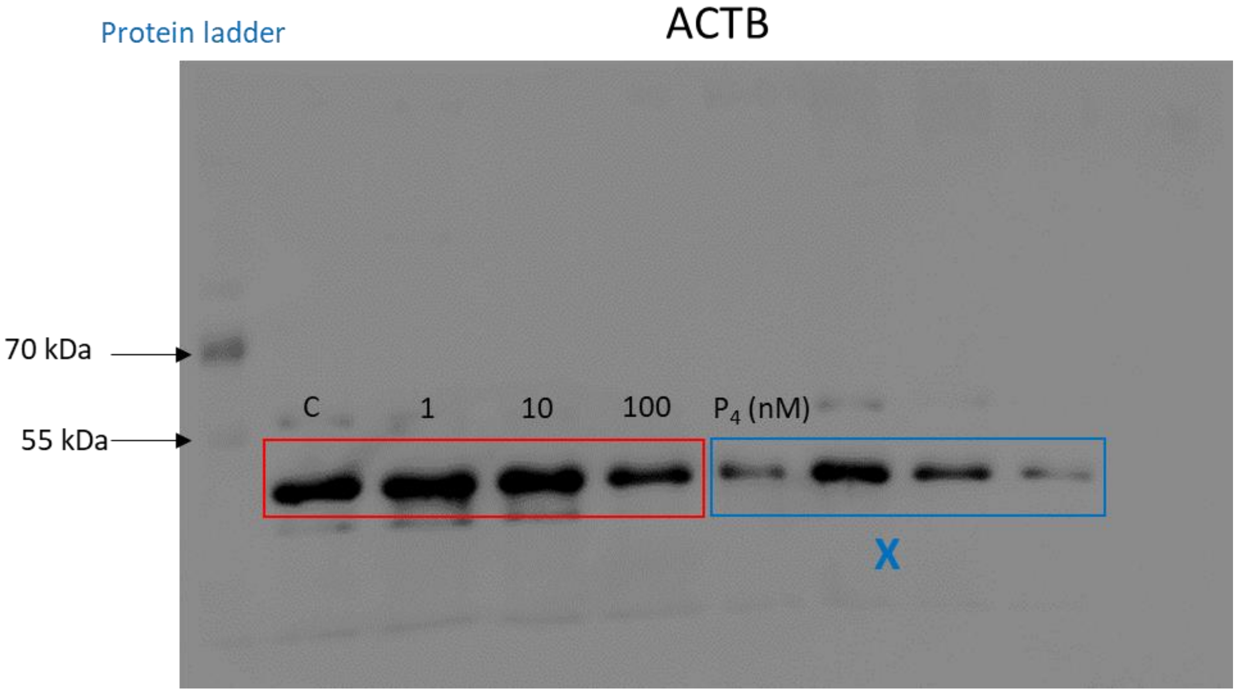

48h:

VISFATIN: 52 kDa

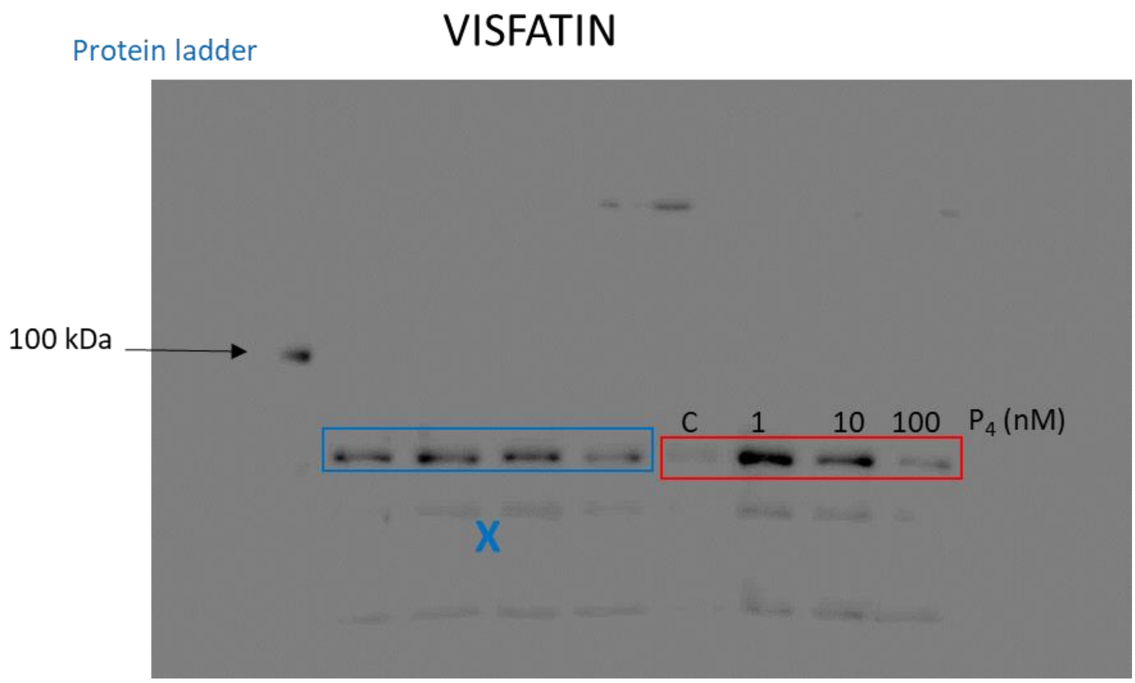

ACTB: 42kDa

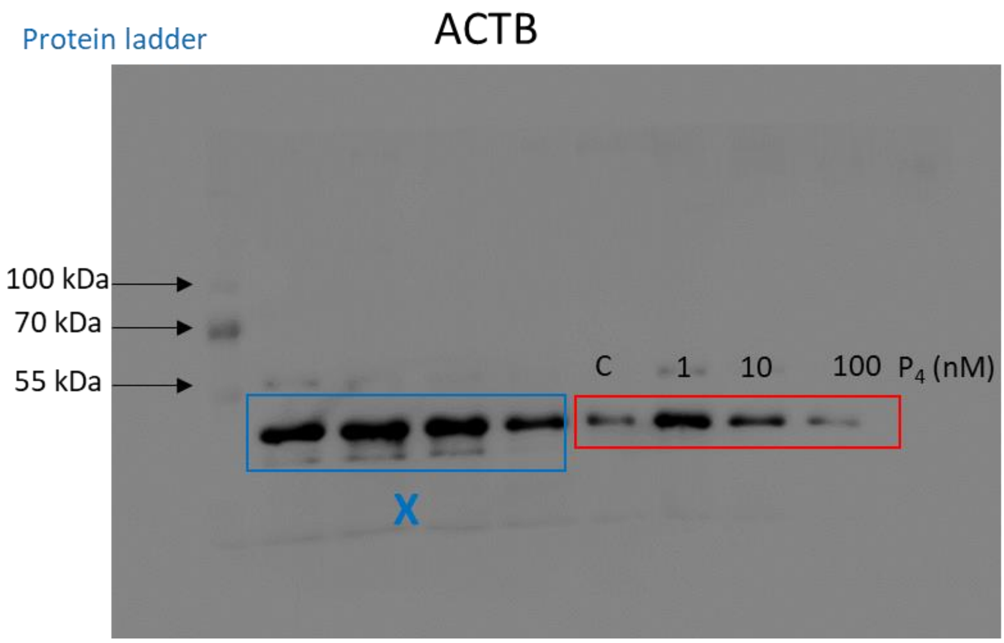

72h:

VISFATIN: 52 kDa

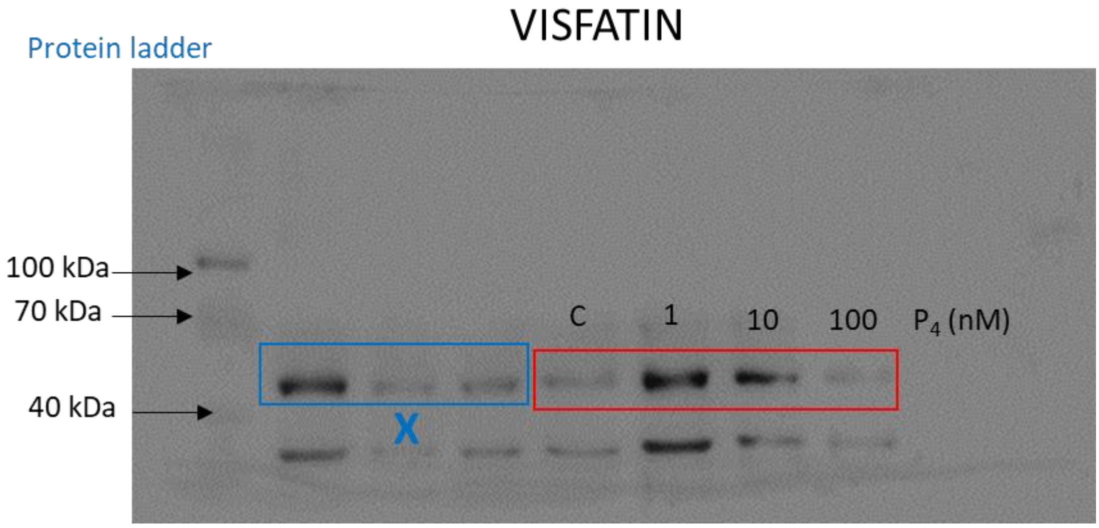

ACTB: 42kDa

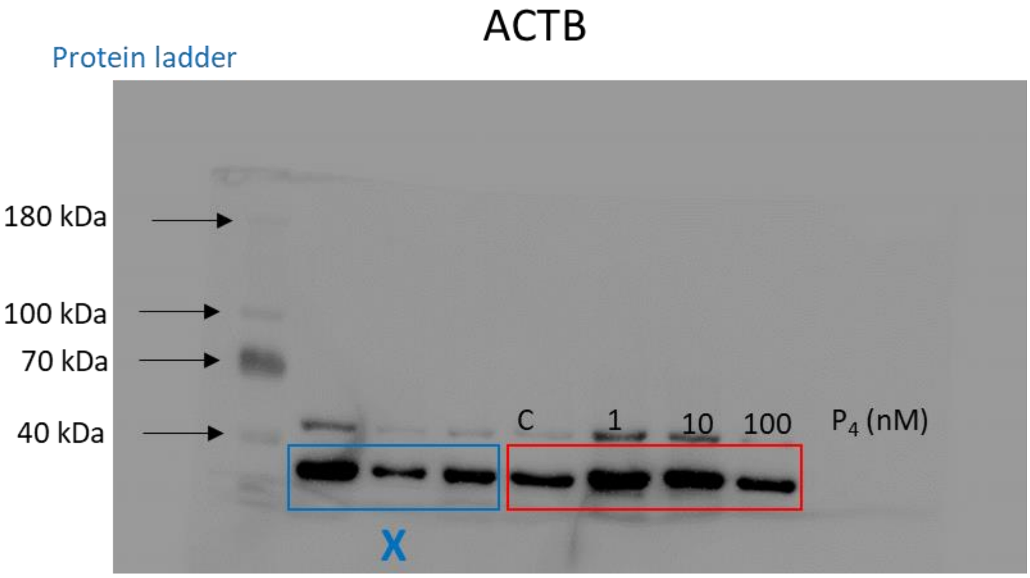

Representative original blots to regulation of visfatin protein expression by E_2 and ACTB JEG-3 (Fig. 5A).

JEG-3

24h:

VISFATIN: 52 kDa

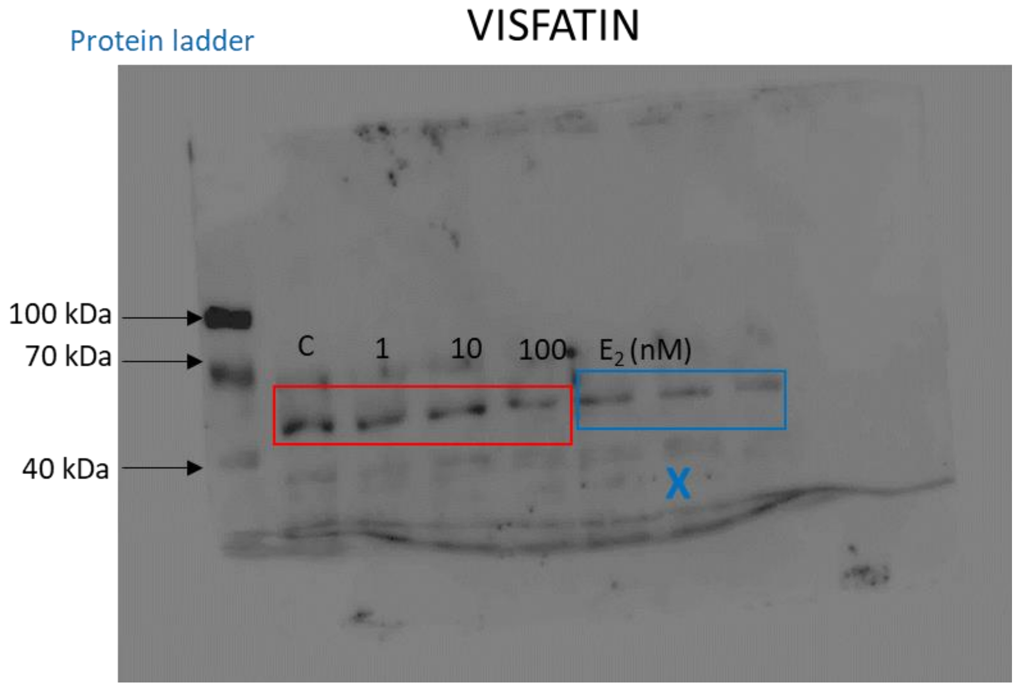

ACTB: 42kDa

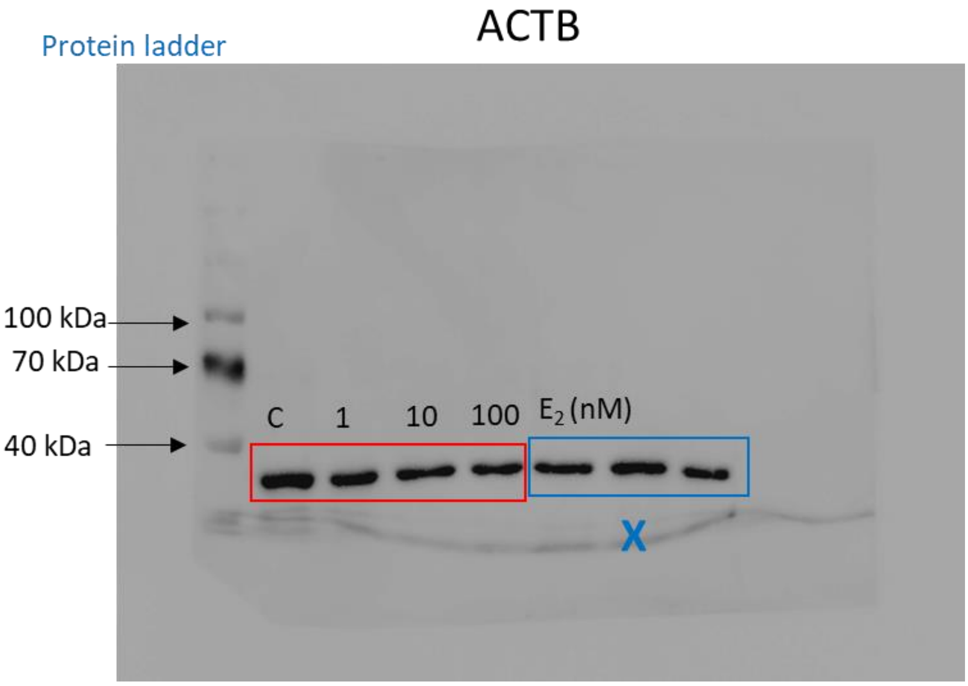

48h:

VISFATIN: 52 kDa

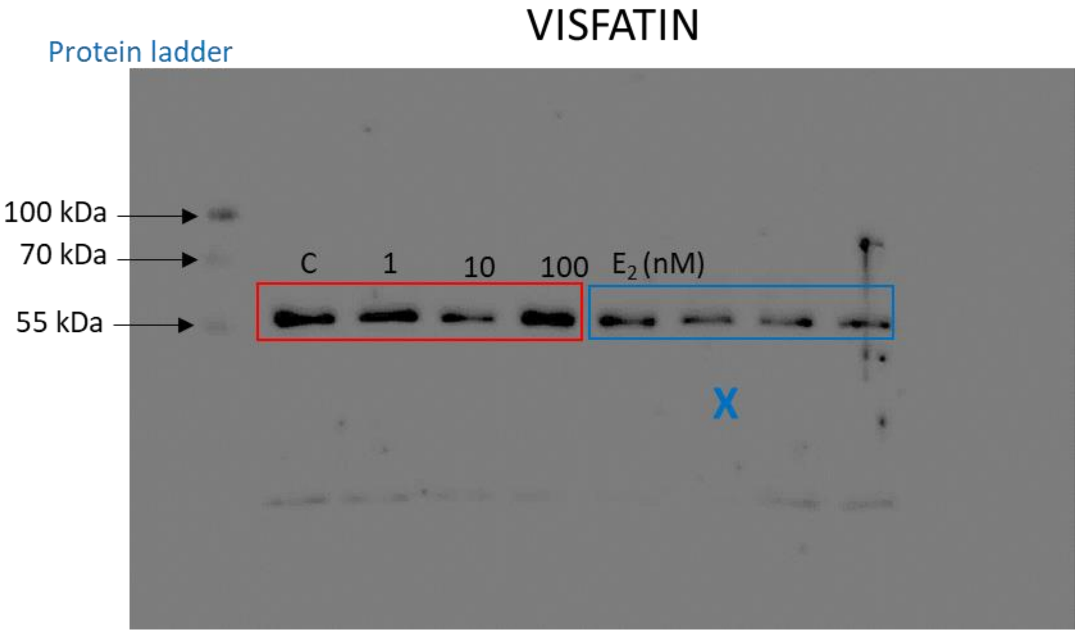

ACTB: 42kDa

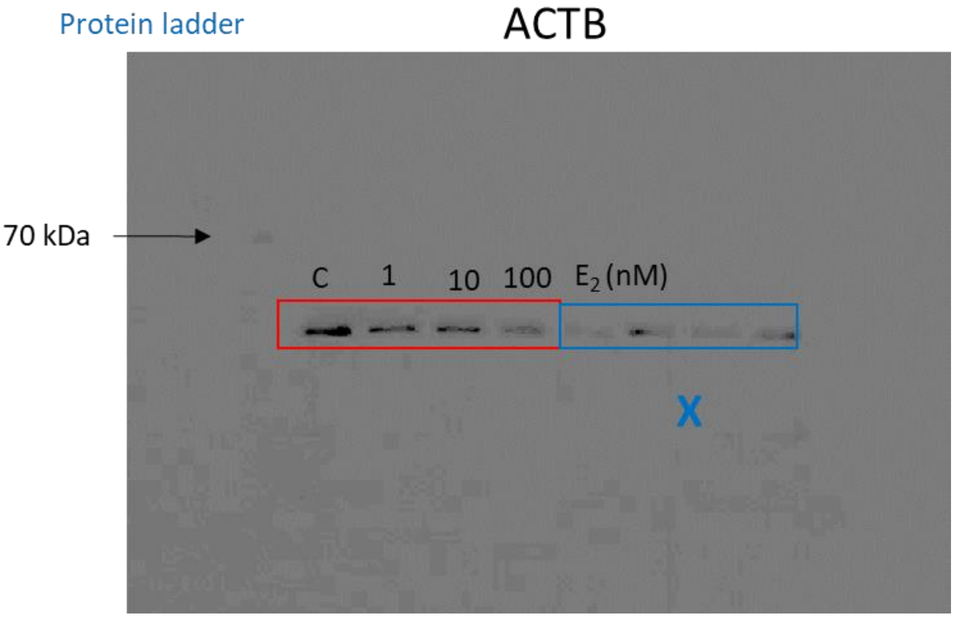

72h:

VISFATIN: 52 kDa

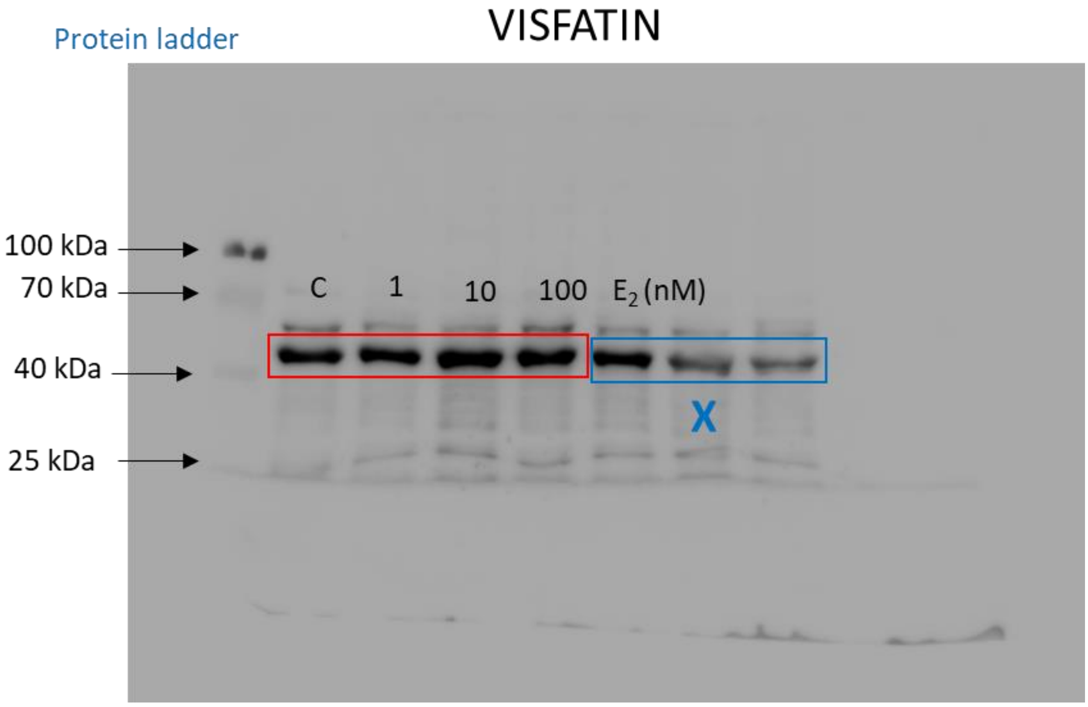

ACTB: 42kDa

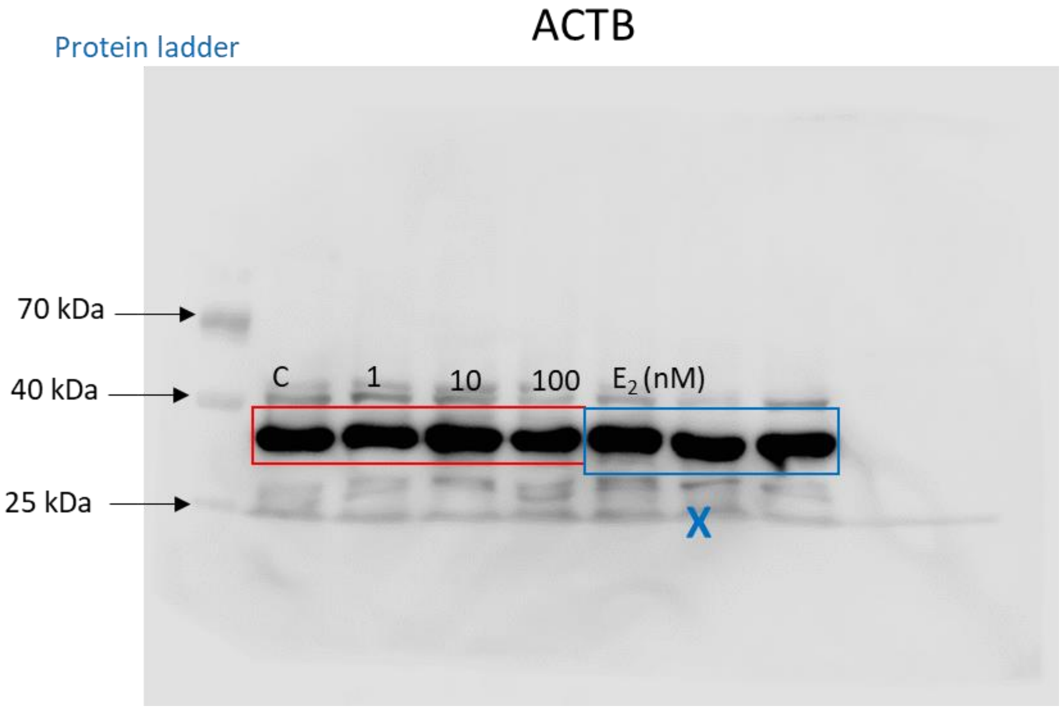

Representative original blots to regulation of visfatin protein expression by hCG and ACTB JEG-3 (Fig. 6A).

JEG-3

24h:

VISFATIN: 52 kDa

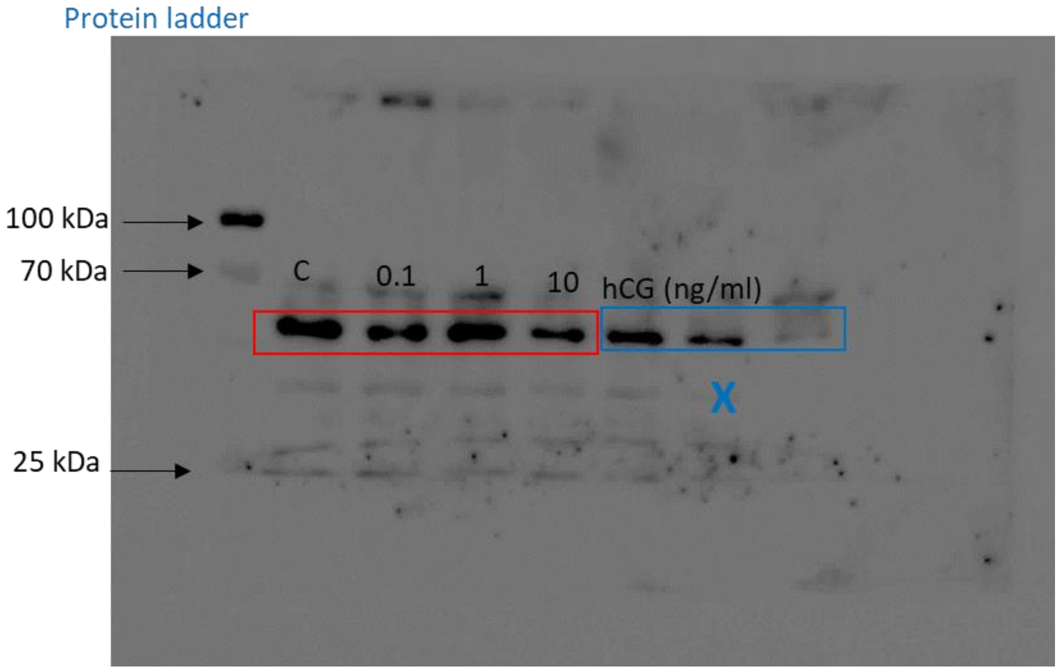

ACTB: 42kDa

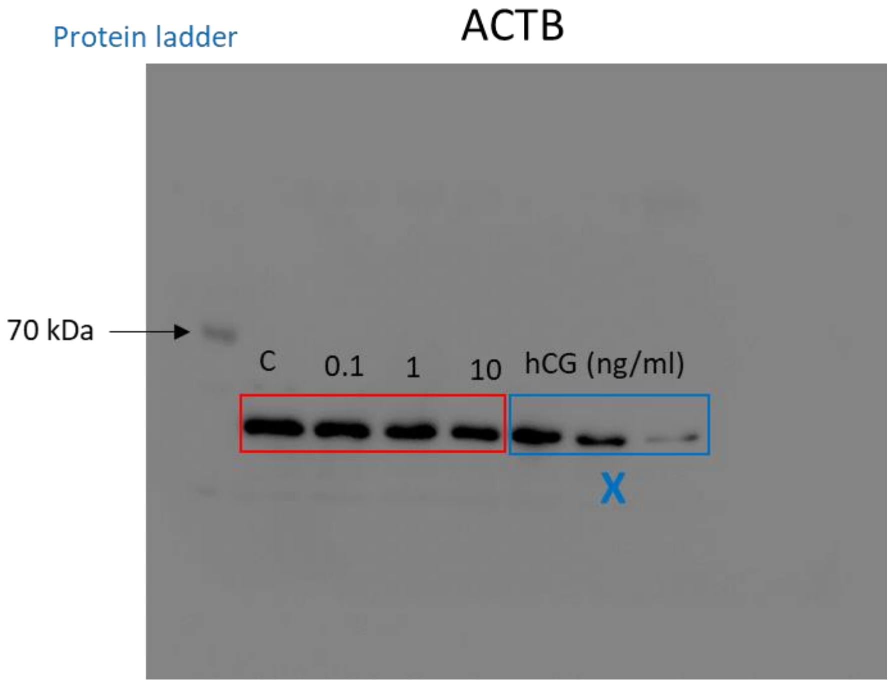

48h:

VISFATIN: 52 kDa

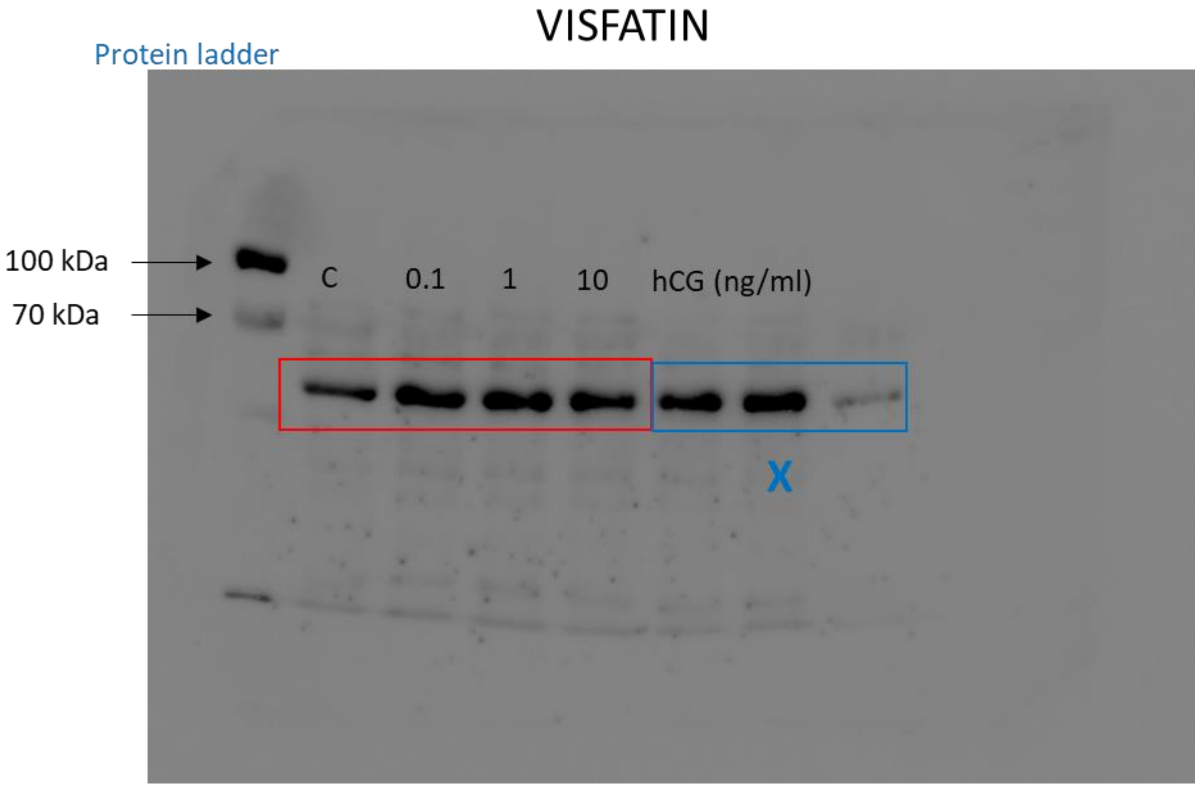

ACTB: 42kDa

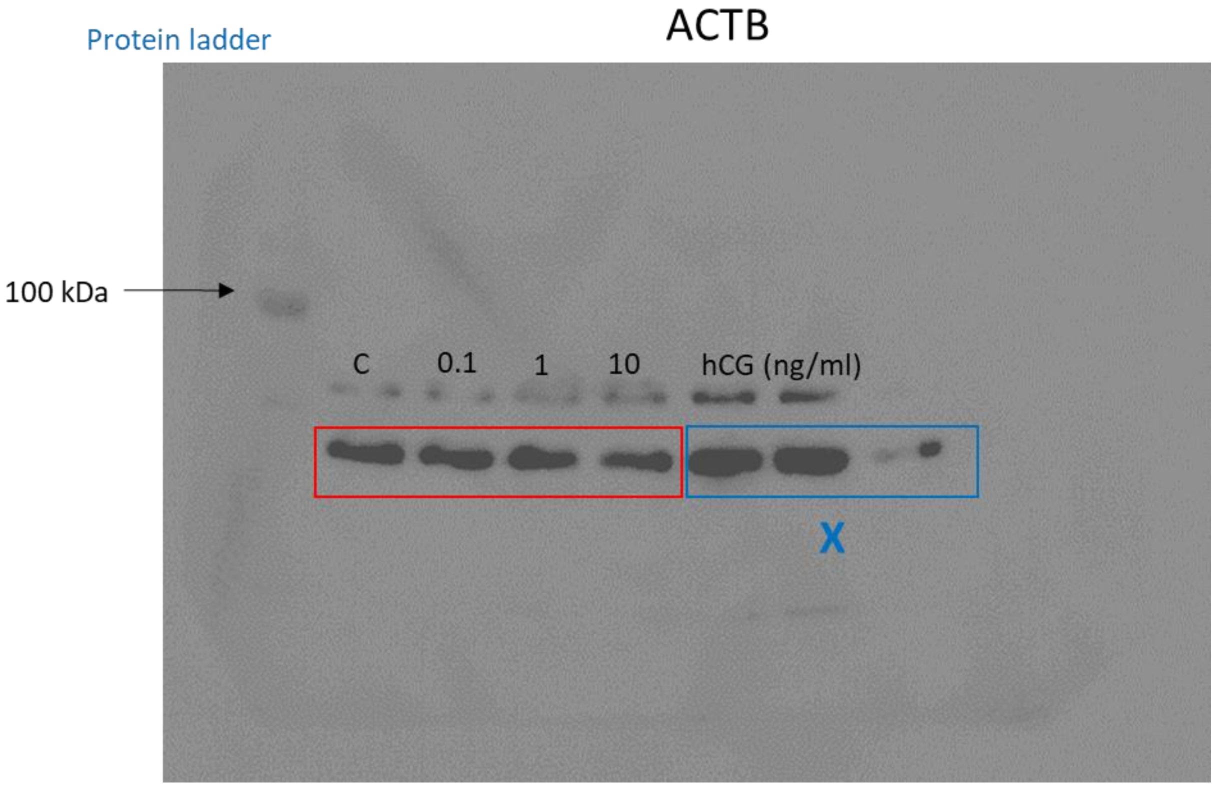

72h:

VISFATIN: 52 kDa

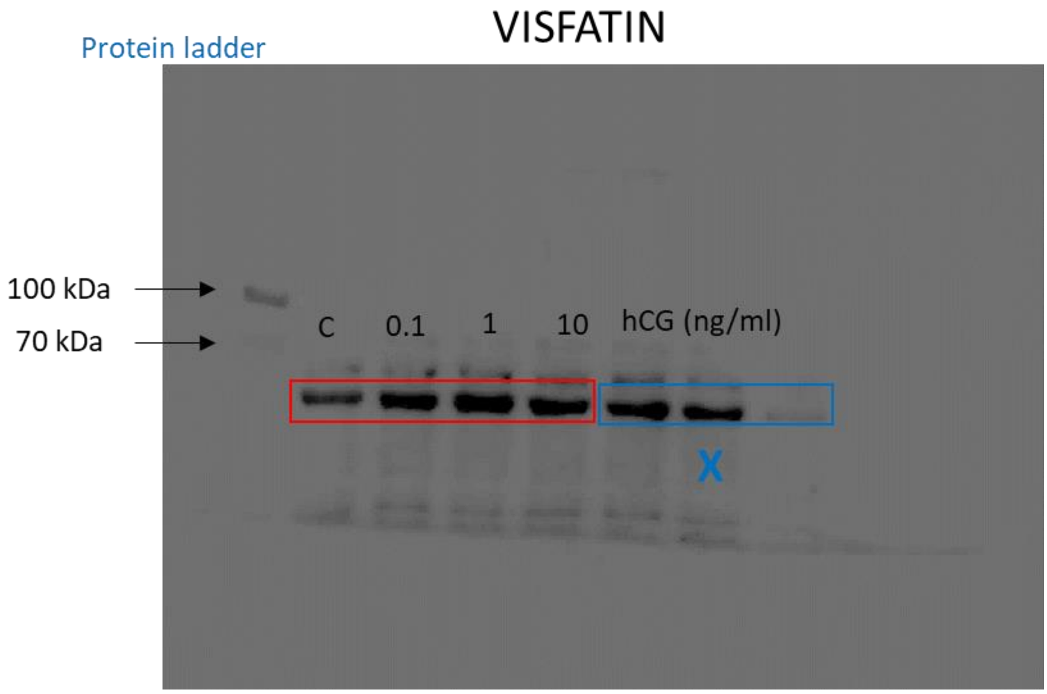

ACTB: 42kDa

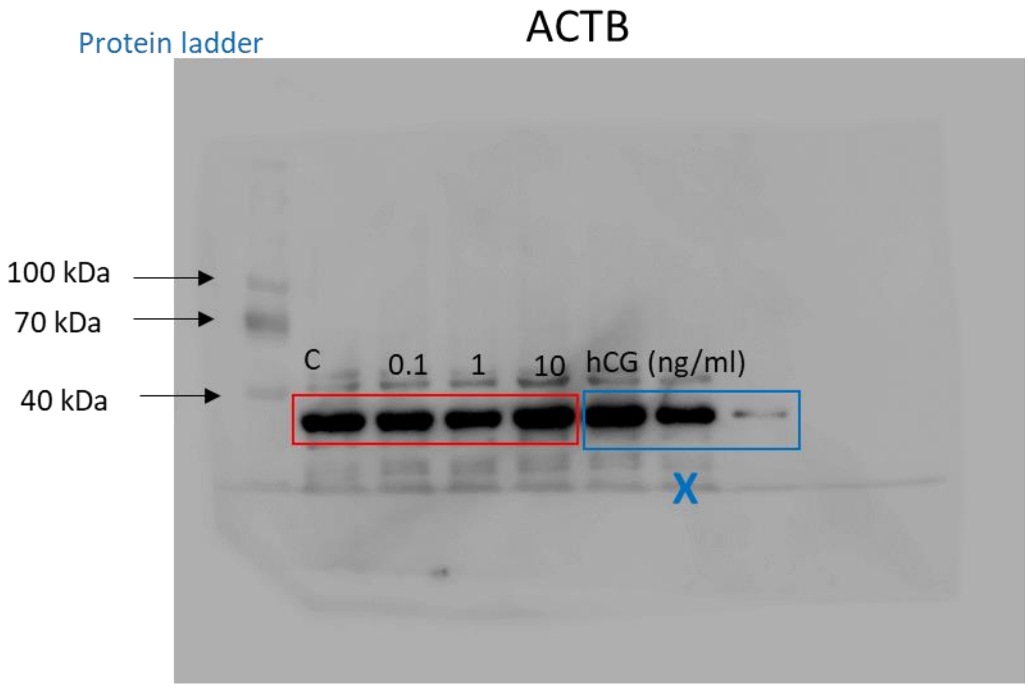

Representative original blots to regulation of visfatin protein expression by INS and ACTB JEG-3 (Fig. 7A).

JEG-3

24h:

VISFATIN: 52 kDa

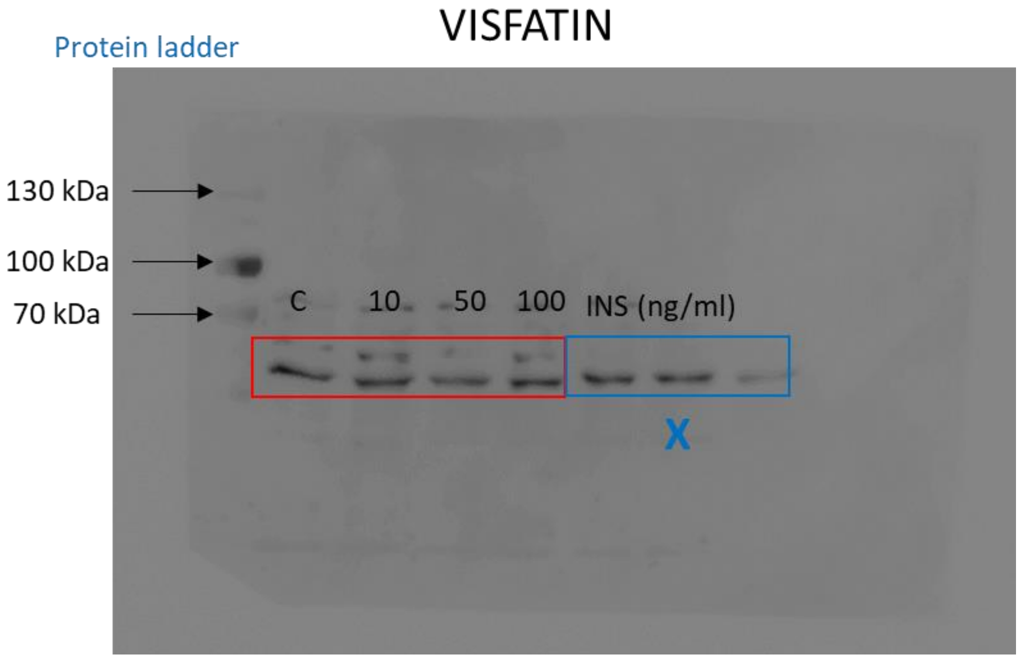

ACTB: 42kDa

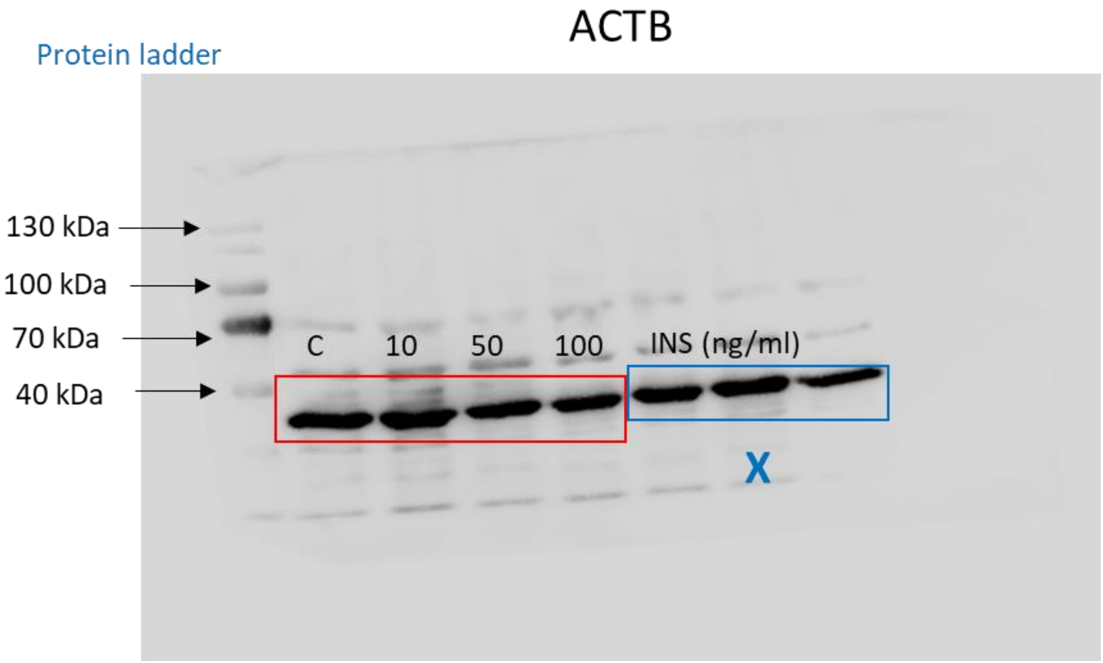

48h:

VISFATIN: 52 kDa

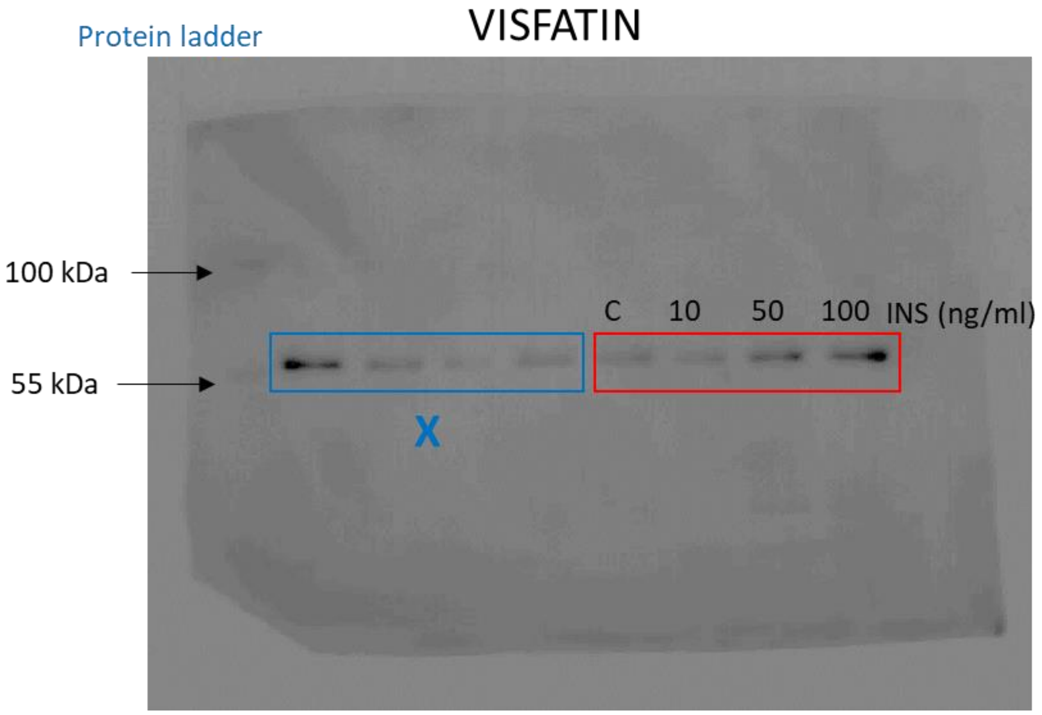

ACTB: 42kDa

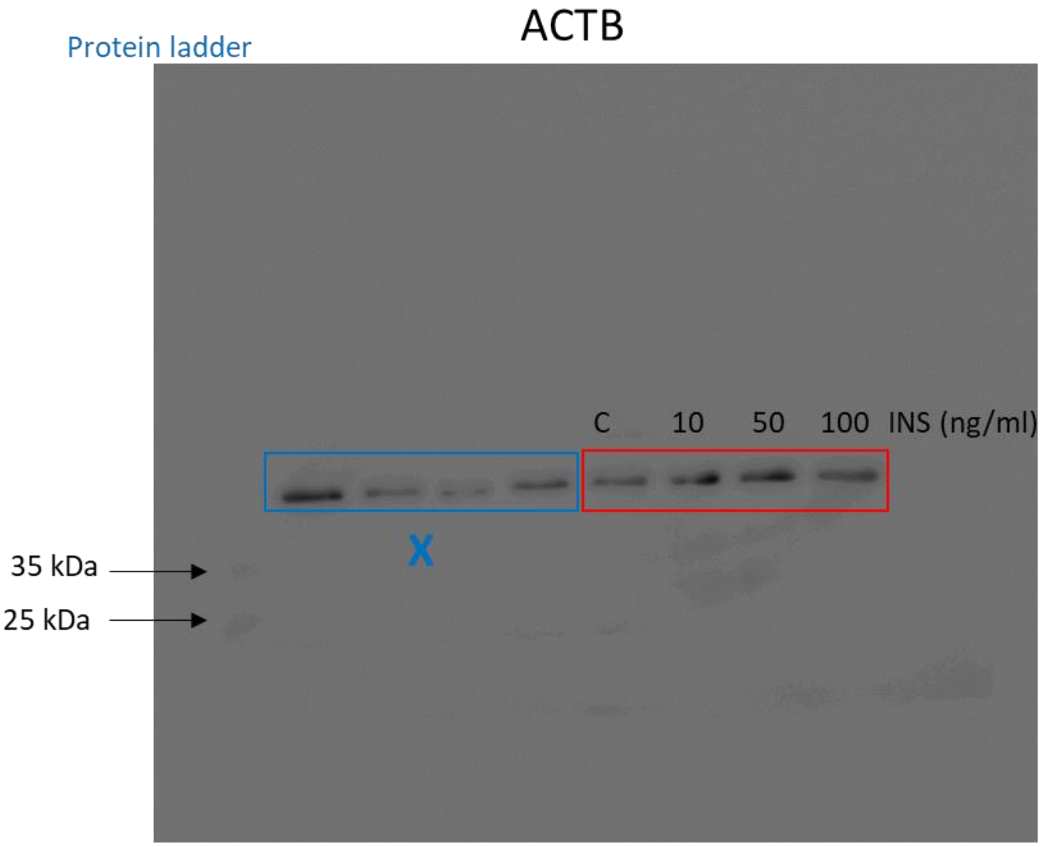

72h:

VISFATIN: 52 kDa

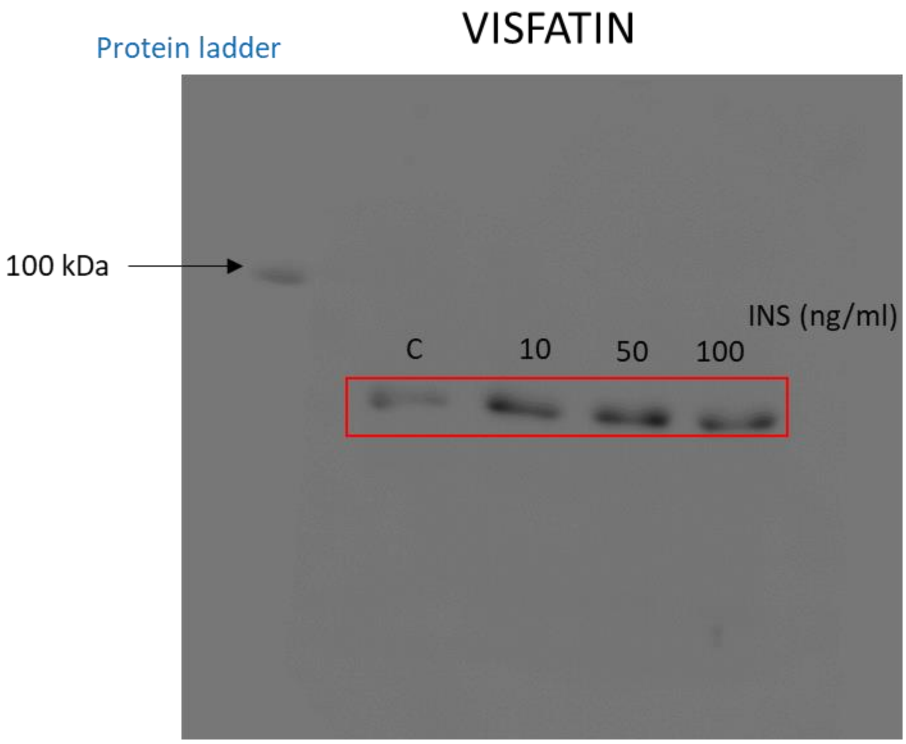

ACTB: 42kDa

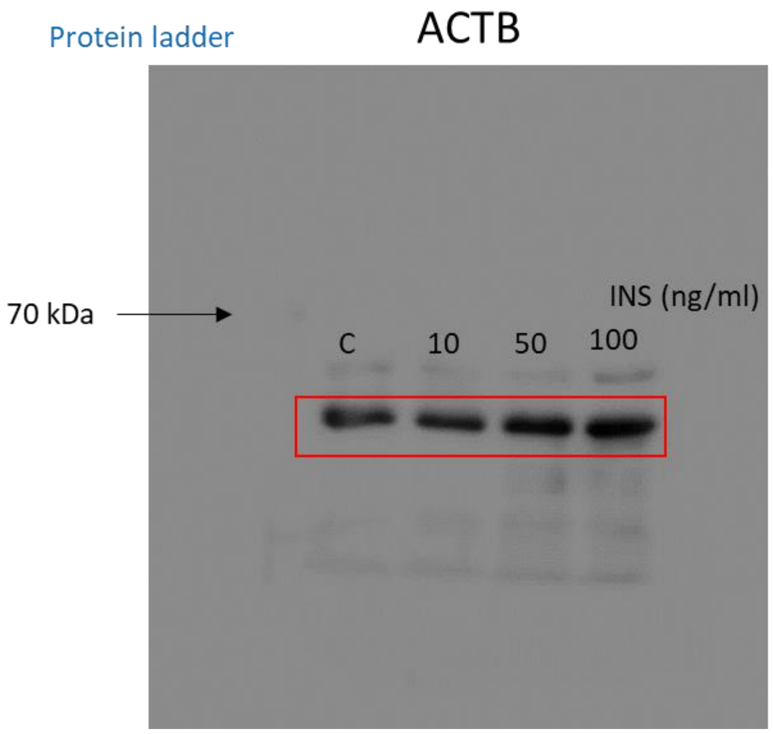

IUGR	Intrauterine growth restriction
PE	Preeclampsia
GDM	Gestational diabetes mellitus
ACTB	β-actin
X	lanes not included in the final figure
	lanes included in the final figure
