# Supplementary material for: Visfatin (NAMPT) expression in human placenta cells in normal and pathological conditions and its hormonal regulation in trophoblast JEG-3 cells
Source: PLoS One. 2024 Sep 18;19(9):e0310389. doi: 10.1371/journal.pone.0310389 (PMC11410215; doi:10.1371/journal.pone.0310389)
Supplement: S2 Raw images — (PDF) [file pone.0310389.s002.pdf]

Representative original Immunohistochemical analysis of visfatin localization in the JEG-3 and BeWo cells (Fig. 2C).

JEG-3

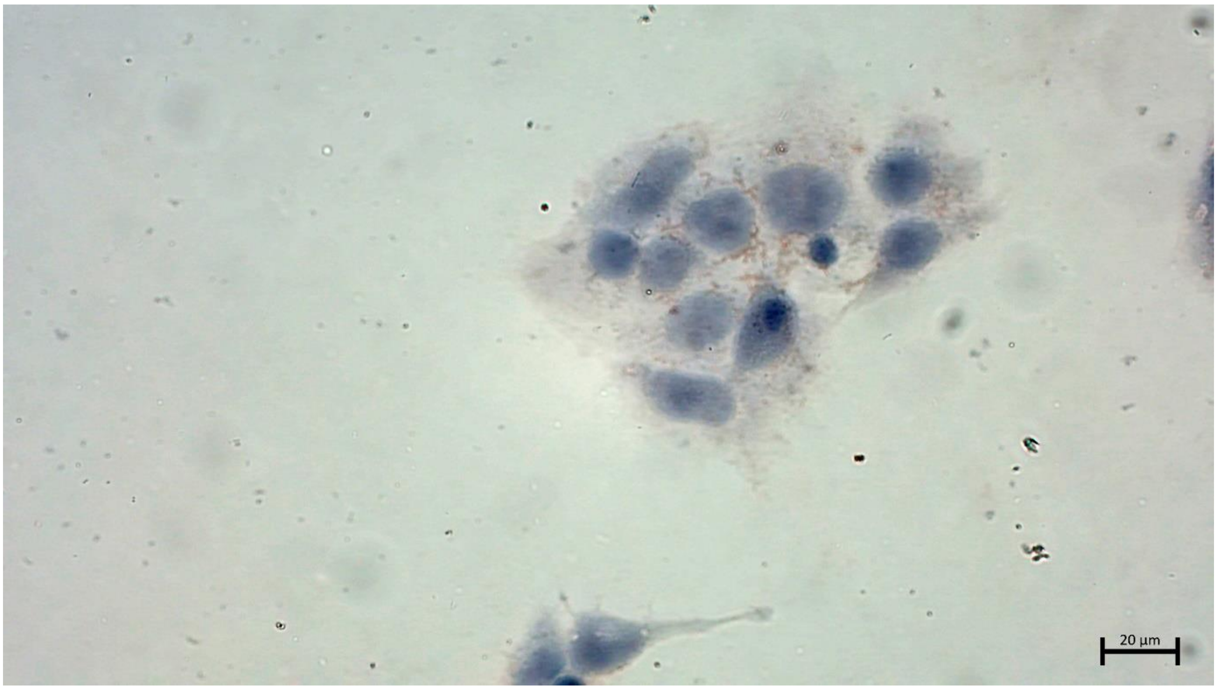

BeWo

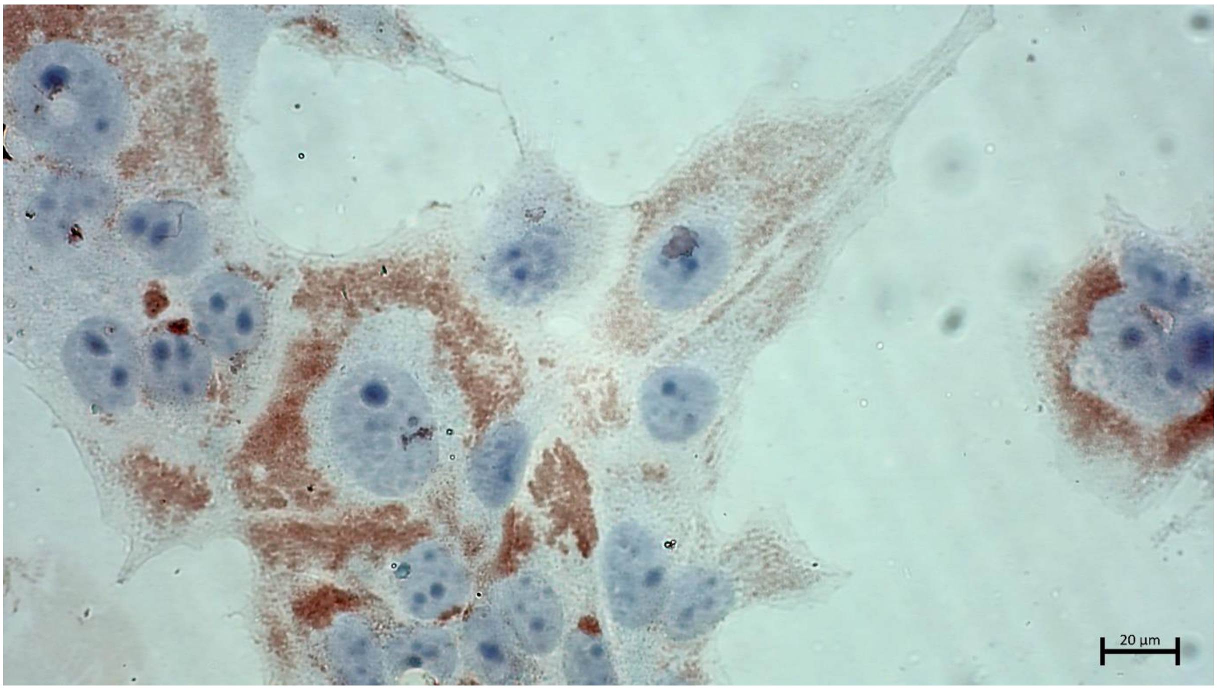

HUMAN PLACENTA

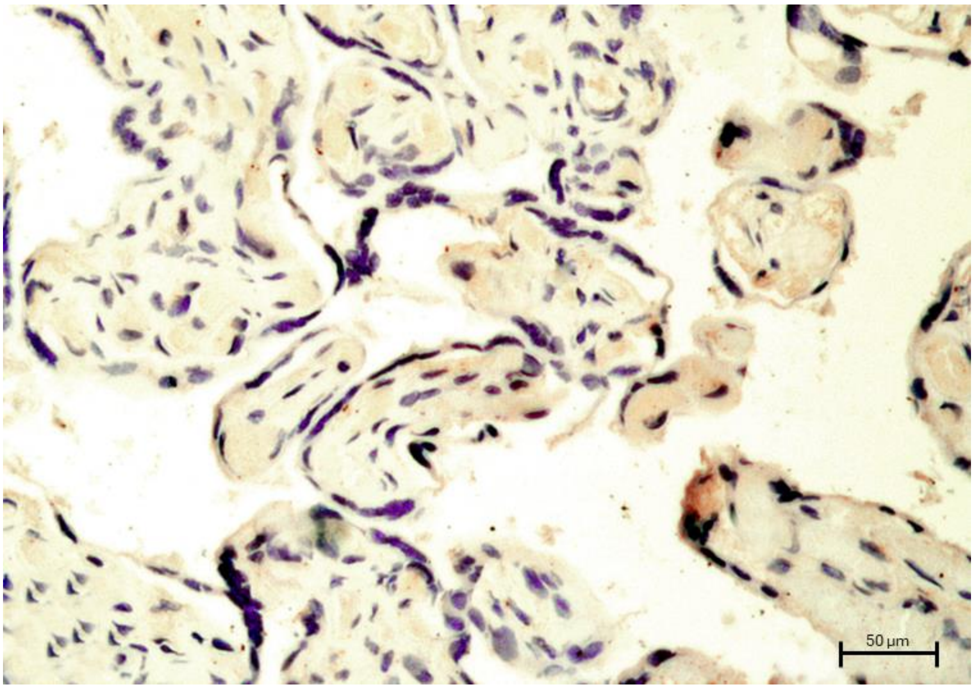

Representative original Immunohistochemical analysis of visfatin localization in the normal, IUGR, PE, GDM placentas (Fig. 3C).

NORMAL PLACENTA

MATERNAL PART

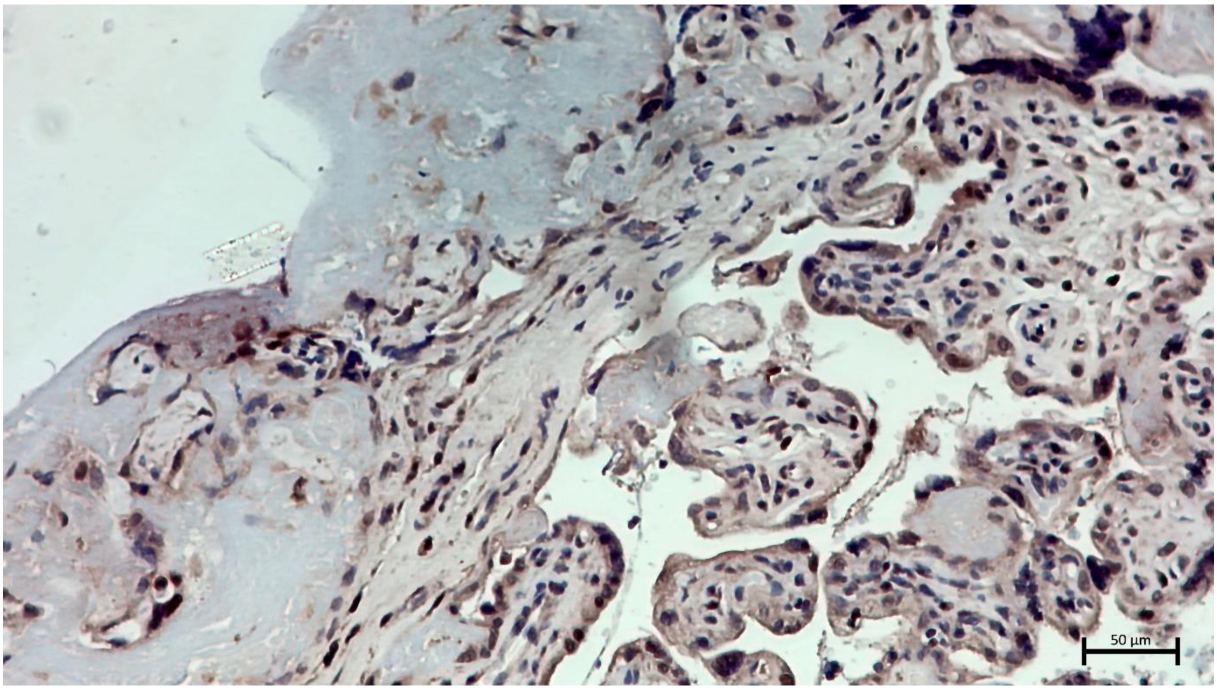

FETAL PART

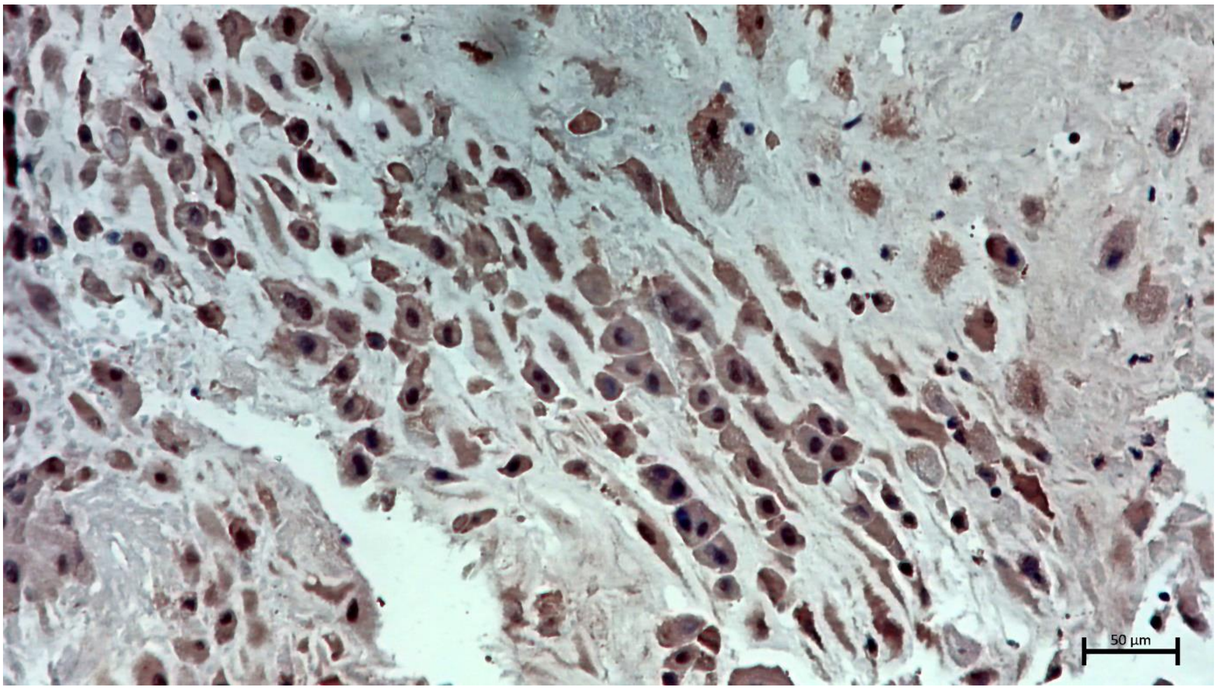

IUGR PLACENTA

MATERNAL PART

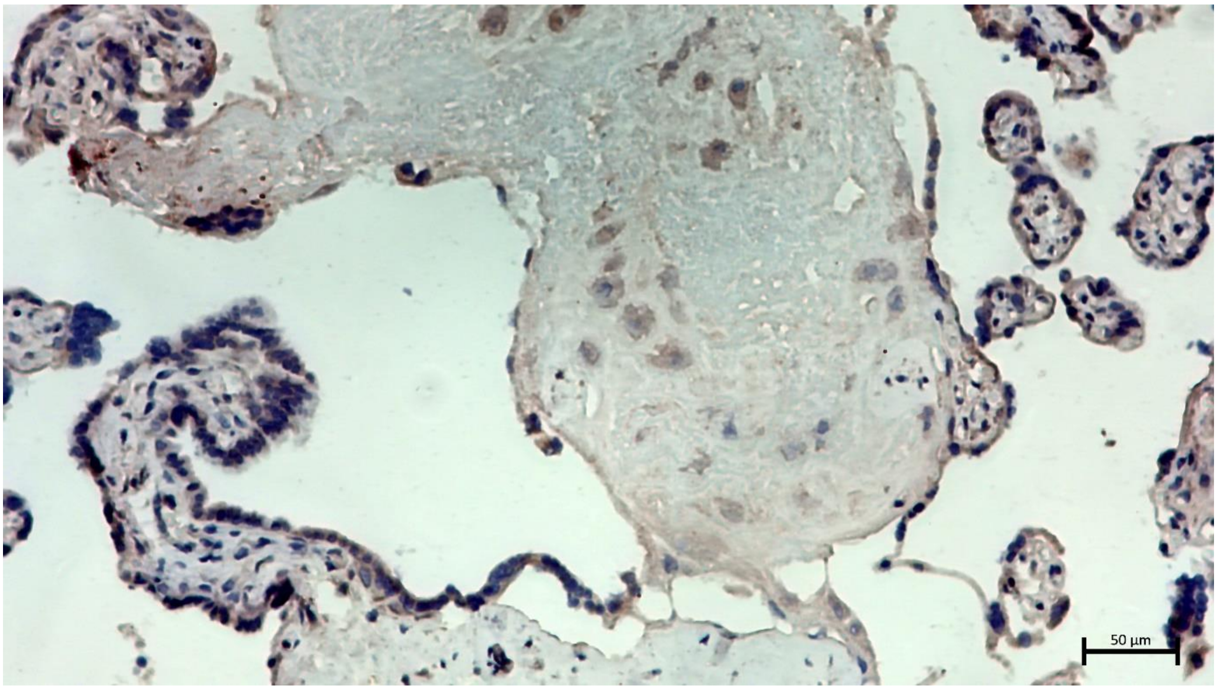

FETAL PART

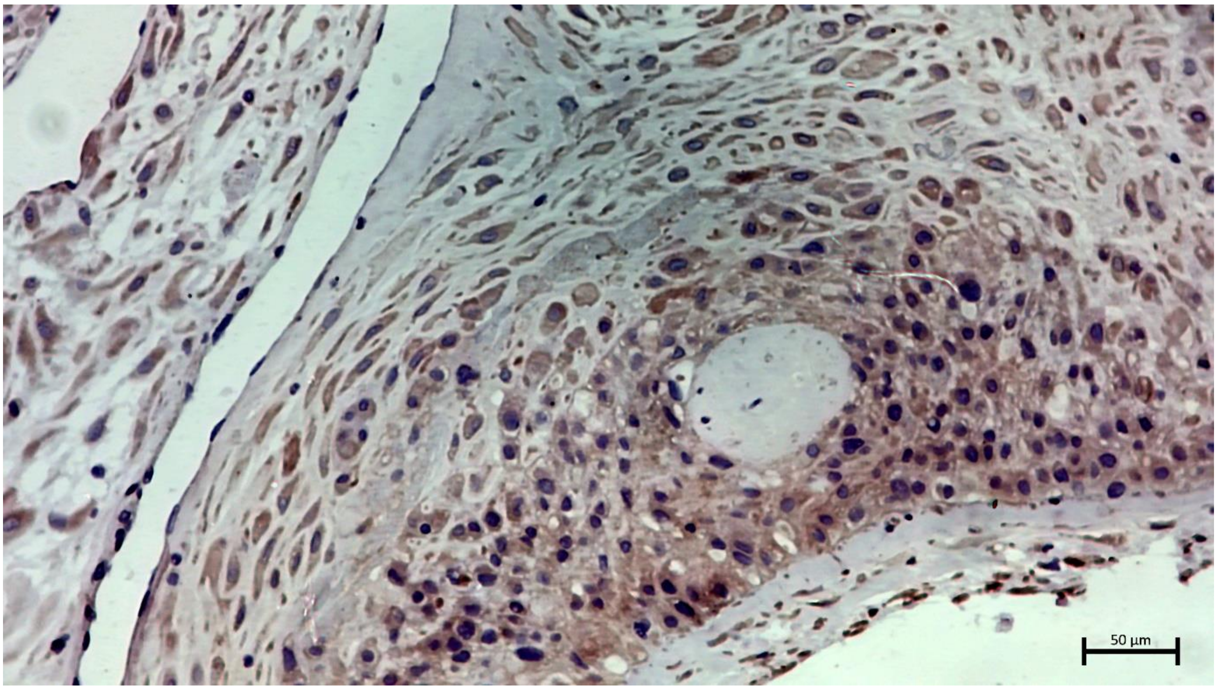

PE PLACENTA

MATERNAL PART

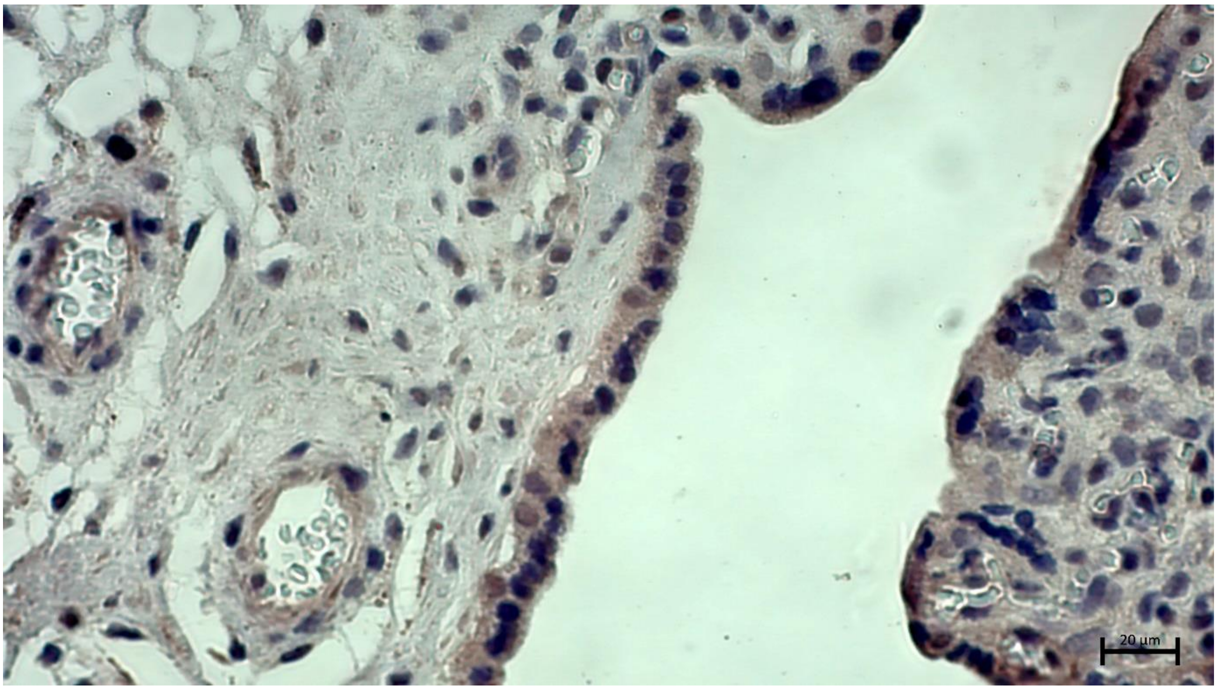

FETAL PART

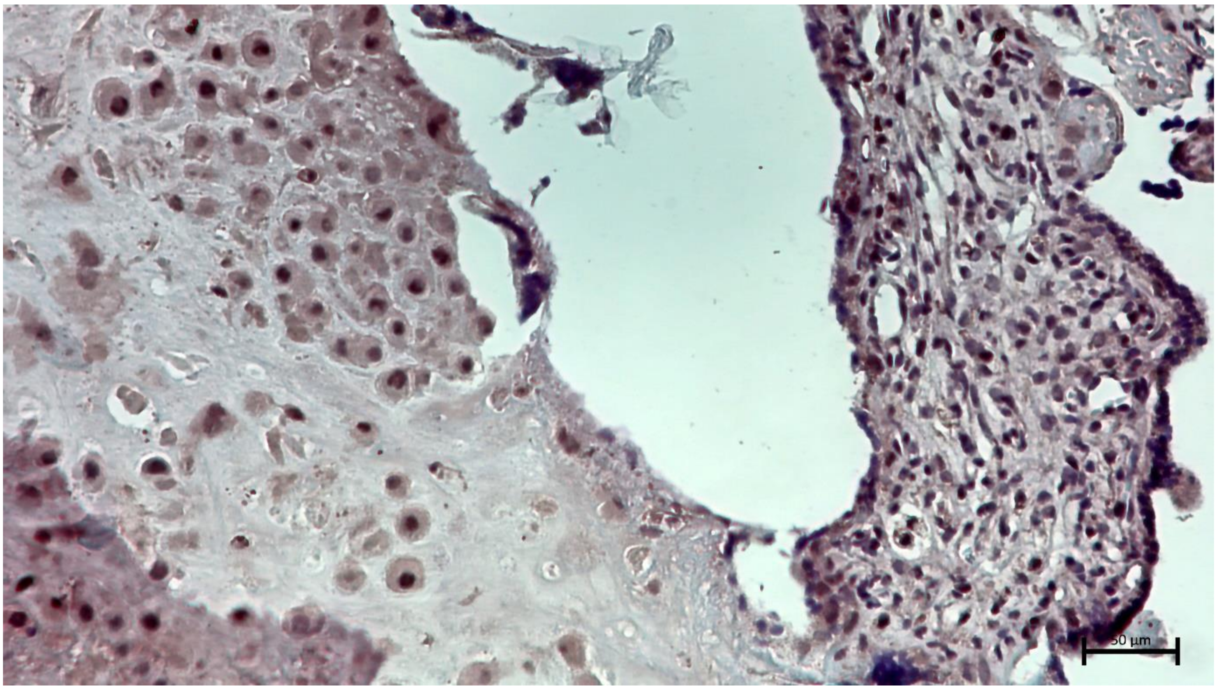

GDM PLACENTA

MATERNAL PART

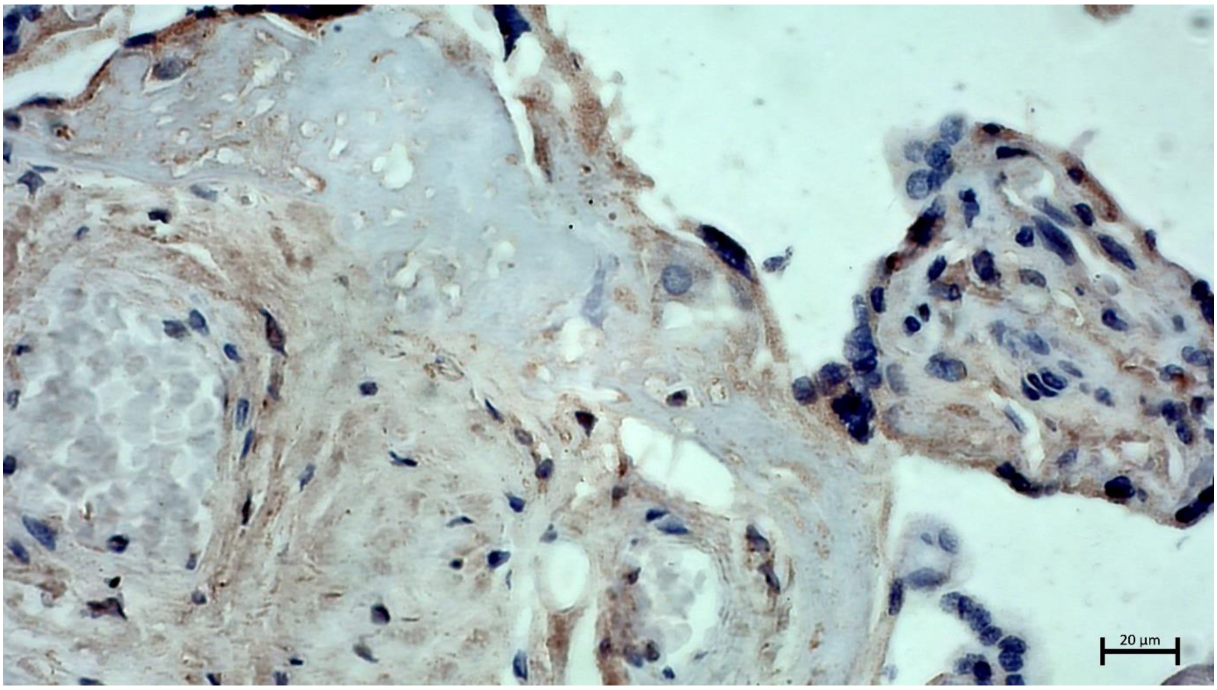

FETAL PART

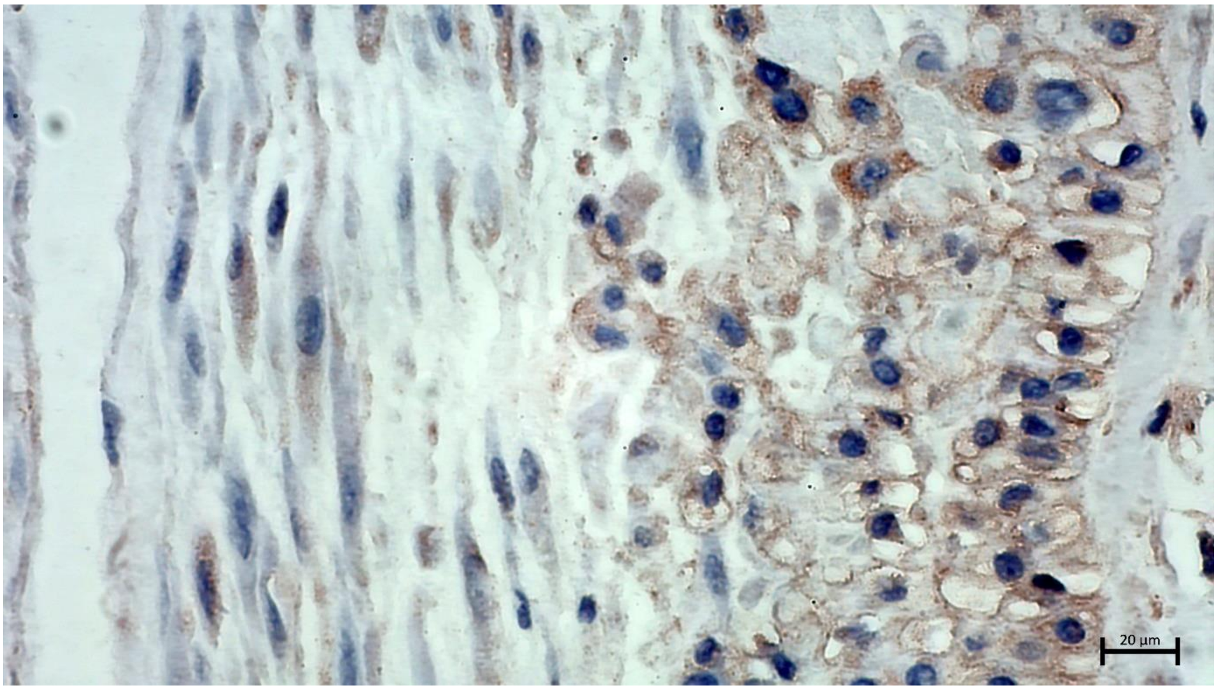

IUGR	Intrauterine growth restriction
PE	Preeclampsia
GDM	Gestational diabetes mellitus
